# The balance between deterministic and stochastic processes in structuring lake bacterioplankton community over time

**DOI:** 10.1111/mec.15538

**Published:** 2020-07-24

**Authors:** Pablo Aguilar, Ruben Sommaruga

**Affiliations:** ^1^ Lake and Glacier Ecology Research Group Department of Ecology University of Innsbruck Innsbruck Austria

**Keywords:** 16S rRNA gene, bacterial community composition, community assembly, mountain lakes, temporal dynamics

## Abstract

One major goal in microbial ecology is to establish the importance of deterministic and stochastic processes for community assembly. This is relevant to explain and predict how diversity changes at different temporal scales. However, understanding of the relative quantitative contribution of these processes and particularly of how they may change over time is limited. Here, we assessed the importance of deterministic and stochastic processes based on the analysis of the bacterial microbiome in one alpine oligotrophic and in one subalpine mesotrophic lake, which were sampled over two consecutive years at different time scales. We found that in both lakes, homogeneous selection (i.e., a deterministic process) was the main assembly process at the annual scale and explained 66.7% of the bacterial community turnover, despite differences in diversity and temporal variability patterns between ecosystems. However, in the alpine lake, homogenizing dispersal (i.e., a stochastic process) was the most important assembly process at the short‐term (daily and weekly) sampling scale and explained 55% of the community turnover. Alpha diversity differed between lakes, and seasonal stability of the bacterial community was more evident in the oligotrophic lake than in the mesotrophic one. Our results demonstrate how important forces that govern temporal changes in bacterial communities act at different time scales. Overall, our study validates on a quantitative basis, the importance and dominance of deterministic processes in structuring bacterial communities in freshwater environments over long time scales.

## 1 INTRODUCTION

It is generally recognized that both deterministic and stochastic processes drive the structure of natural microbial communities (Caruso et al., 2011; Chase, 2010; Chase & Myers, 2011; Chen et al., 2019; Leibold et al., 2004; Vellend, 2010; Zhou & Ning, 2017). Deterministic processes (niche‐based processes) are the result of the selection imposed by the abiotic environment and both antagonistic and synergistic species interactions (Stegen, Lin, Konopka, & Fredrickson, 2012). In contrast, stochastic processes (neutral‐based processes) include chance colonization and random extinction (Chase & Myers, 2011). Further, ecological drift can also result from fluctuations in population sizes due to chance events. Based on the phylogenetic turnover in community composition, deterministic processes are divided into homogeneous selection (i.e. consistent environmental factors primarily cause low compositional turnover) and variable selection (high compositional turnover primarily caused by shifts in environmental factors), whereas stochastic processes are divided into homogenizing dispersal (low compositional turnover caused by high dispersal rates) and dispersal limitation (high compositional turnover caused by a low rate of dispersal) (Stegen et al., 2012, 2013; Stegen, Lin, Fredrickson, & Konopka, 2015).

Different studies on terrestrial and aquatic environments have proposed that bacterial community assembly is governed primarily by deterministic processes (Stegen et al., 2012; Vanwonterghem et al., 2014; Wang et al., 2013; Zhao et al., 2017), although the relative contribution of different processes has been quantified only recently (Liu et al., 2020; Logares et al., 2018; Stegen et al., 2013; Vass, Székely, Lindström, & Langenheder, 2020; Yan et al., 2017). Indeed, the first quantification of the relative importance of different processes responsible for microbial community assembly considered only spatial scales (Stegen et al., 2013). This study demonstrated that depending on the depth and type of sediment, the importance of deterministic and stochastic processes change, and that drift alone explains ca. 25% of the spatial turnover in community composition. Studies on how those ecological processes contribute to the assembly of lake microbial communities over time are scarce and focused on either long‐term (e.g. decade, century) or short‐term (e.g., 5 weeks) time scales (Liu et al., 2020; Vass et al., 2020; Yan et al., 2017), yet there is a need to understand how deterministic and stochastic processes potentially change in one ecosystem at different time scales (Jia, Dini‐Andreote, & Falcão Salles, 2018; Ladau & Eloe‐Fadrosh, 2019).

Studies on temporal bacterial dynamics lasting for >1 year have mainly been done in marine systems, whereas in freshwater ecosystems, most have lasted ≤1 year. Interestingly, studies on marine microbial communities have shown that variability patterns are predictable at the daily, seasonal and interannual scale of variation (Fuhrman, Cram, & Needham, 2015). In lakes, it has also been described that a high temporal variability of bacterioplankton exists with recurrent seasonal patterns and annual periodicity (Li et al., 2015; Shade, Caporaso, Handelsman, Knight, & Fierer, 2013; Shade et al., 2007; Van Der Gucht et al., 2001). However, it is unclear how predictable those patterns are at different time scales and what ecological processes explain the assembly of communities. Some studies using high‐throughput sequencing have shown the importance of long‐term sampling to achieve a complete understanding of the bacterial dynamics in freshwater ecosystems (Linz et al., 2017; Obertegger, Bertilsson, Pindo, Larger, & Flaim, 2018).

In the present study, we hypothesized that the temporal variability of lake bacterial communities differs across various time scales and trophic states. We expected that the bacterial community of an oligotrophic lake would be more stable (Yannarell, Kent, Lauster, Kratz, & Triplett, 2003), whereas in a more productive system, bacterial communities would have higher diversity and lower similarity over time (Dai et al., 2017; Llirós et al., 2014). Thus, we first tested whether the bacterial community composition from an oligotrophic alpine lake differed when assessed at annual and short‐term sampling scales (weekly and daily). Second, we tested whether temporal variability was less variable in an oligotrophic lake than in a mesotrophic one located in the same region. Finally, we hypothesized that the balance of ecological processes would change when different time scales are considered. To assess the diversity and composition of the bacterial community in each lake and to estimate the contribution of the different processes leading to its assembly, we based our analysis on clustering the sequences (reads) into amplicon sequence variant (ASV) with the resolution of a single‐nucleotide difference (Callahan et al., 2016). As shown in the literature, this is a more powerful and reproducible method than using OTUs (Callahan, McMurdie, & Holmes, 2017).

## 2 MATERIALS AND METHODS

### 2.1 Study area and sampling

Sampling was done in two mountain lakes located in the Austrian Alps having different trophic state. Gossenköllesee (hereafter, GKS; 47°13′N, 11°00′E) is a 1.7 ha alpine (i.e., located above tree line at 2,417 m a.s.l.) oligotrophic lake with a maximum depth of 9.9 m (Sommaruga & Psenner, 1995). This lake is holomictic/dimictic and ice‐covered for up to 7 months (Rofner, Sommaruga, & Pérez, 2016). Piburgersee (hereafter, PIB; 47°11′, 10°53′E) is a 13.4 ha, mesotrophic subalpine (913 m a. s. l.) lake with a maximum depth of 24.6 m. Usually, this lake is ice‐covered from early December to April (Niedrist, Psenner, & Sommaruga, 2018). Both lakes are part of the Long‐Term Socio‐Ecological Research platform Tyrolean Alps and GKS is also part of the Global Lake Ecological Observatory Network (GLEON).

The annual‐scale sampling consisted of composite water samples (i.e., same volume pooled from every single depth; GKS: 0.5, 2, 4, 6, and 8 m; PIB: 0.5, 3, 6, 9, 12, 15, 18, 21, and 24 m) collected monthly between November 2014 and December 2016 in GKS (*n* = 26), as well as between December 2014 and December 2016 in PIB (*n* = 25). All samples were collected with a modified Schindler‐Patalas sampler from a boat placed over the deepest area of the lake. The short‐term sampling was done only in GKS during 2016 and consisted of composite samples (1,000 ml) collected in triplicates (*n* = 9) taken at weekly intervals (8, 16 and 21 August) and composite samples in triplicates (*n* = 27) taken twice per day (at 7 a.m. and 7 p.m. local time) between 16 and 21 August. In addition, samples at single depths were collected once in GKS (5 samples; 0.5, 2, 4, 6, and 8 m) and PIB (9 samples; 0.5, 3, 6, 9, 12, 15, 18, 21 and 24 m) during late May 2016 (GKS) and early June 2016 (PIB), before the thermal mixing period occurred between late June and early July, respectively.

Water samples were kept in cold boxes, and afterwards (within ca. 3 h), they were filtered onto 0.22‐µm pore size filters (47 mm, Millipore GPWP). In GKS, the volume filtered ranged from 800 to 1,000 ml, whereas in PIB, it was between 800 and 900 ml. Filters were placed in Eppendorf tubes with RNAlater (Qiagen, Germantown, MD) and maintained at −20°C until DNA extraction took place.

### 2.2 Environmental data

In situ monthly measurements of water temperature were done with a thermometer placed inside the water sampler in GKS and with a multiparameter probe (EXOSonde2; YSI) in PIB. The specific electrical conductivity (25°C) and pH were measured in the laboratory (within ca. 3 h) with a portable conductivity meter (LF 196, WTW) and a pH meter (Orion930, Orion Ross‐Electrode), respectively. Additionally, monthly samples were also collected in parallel for water chemical analyses. Major anions (nitrate, chloride and sulphate) and cations (potassium, sodium, calcium and magnesium) were measured by ion chromatography (Dionex ICS‐1100/1000). Ammonium was measured by the Indophenol blue method (Wagner, 1969). Samples were also collected in precombusted (4 h at 450°C) glass bottles for the analysis of dissolved organic carbon (DOC) and dissolved nitrogen (DN). These samples were filtered in situ through precombusted GF/F filters (Whatman). The filtrate was acidified with HCl to pH 2 and analysed later with a Shimadzu TOC‐Vc series instrument equipped with a total nitrogen module. Calibration for DOC analysis was done with potassium hydrogen phthalate, whereas for the DN, it was done with potassium nitrate. Three to five subsamples were analysed for each sample and for a consensus reference material (CRM) for DOC (batch 5 FS‐2005:0.57 mg; provided by RSMAS/MAC, University of Miami) that was run in parallel on each occasion. Results differed from the CRM given value by 5%, and the coefficient of variation among subsamples was <2%. Total dissolved phosphorus (DP) concentrations were estimated by the molybdenum blue method (Vogler, 1966). The measurement of chlorophyll‐*a* as a proxy for phytoplankton biomass was done as described in Tartarotti and Sommaruga (2006), and the equation of Lorenzen (1967) was used to calculate its concentration. Dissolved oxygen was measured only in PIB by the Winkler method.

### 2.3 DNA extraction and sequencing

Genomic DNA was extracted using the PowerWater DNA isolation kit (Mo Bio Laboratories Inc.) following the manufacturer's protocol. The concentration and quality of DNA were measured with a NanoDrop spectrophotometer (NanoDrop 8000, Thermo Scientific). DNA was used as a template for the V4‐V5 region amplification of the 16S SSU rRNA with the primers 515F‐Y (5'‐GTGYCAGCMGCCGCGGTAA‐3' and 926R (5'‐CCGYCAATTYMTTTRAGTTT‐3') (Parada, Needham, & Fuhrman, 2016). Sequencing was done at LGC Genomics (Berlin, Germany) using the Illumina Miseq platform. Briefly, each PCR was carried out with 1–10 ng of DNA extract (total volume 1 µl), 15 pmol of each forward primer and reverse primer (in 20 µl volume of 1× MyTaq buffer containing 1.5 units MyTaq DNA polymerase (Bioline)), 2 µl of BioStabII PCR Enhancer (Sigma) and additionally 0.2 µl of DNase (Articzymes). The program was set to 20 cycles, using the following parameters: 1 min 96°C predenaturation; 96°C for 15 s, 50°C for 30 s, 70°C for 90 s. For this reaction, barcoded primers, 515F‐Y/926R (Parada et al., 2016), were added. DNA concentration of amplicons of interest was determined by gel electrophoresis. About 20 ng amplicon DNA of each sample was pooled for up to 48 samples having different barcodes. The amplicon pools were purified with one volume AMPure XP beads (Agencourt) to remove primer dimers and other small mispriming products, followed by an additional purification step on MinElute columns (Qiagen). About 100 ng of each purified amplicon pool DNA was used to construct Illumina libraries using the Ovation Rapid DR Multiplex System 1–96 (NuGEN). Illumina libraries were pooled and size selected by preparative gel electrophoresis. Sequencing was done on an Illumina MiSeq using V3 Chemistry (Illumina). Raw amplicon reads were deposited in the Sequence Read Archive (SRA) of NCBI under Accession no SRP167155.

### 2.4 Amplicon data processing

Raw amplicons from 101 samples were analysed using the R package DADA2, version 1.8.0 (Callahan et al., 2016; R Core Team, 2018). Briefly, after inspection of read quality profiles, the forward reads were trimmed to 240 bases and the reverse ones to 180 bases. All reads containing more than two expected errors were removed. The error rates were learned from a subset of 1,046,328 reads. These error rates were used to infer the ASVs. The forward and reverse reads were merged to obtain the full denoized sequence of ASVs. Denoized sequences with one or more mismatch in the overlap region were removed. The chimeras were removed using “removeBimeraDenovo”. Finally, ASVs were classified using the Silva reference data set version 132 and a table with read counts and taxonomy of all ASVs was constructed. Samples with <10,000 sequences and sequences classified as Archaea, NA (unknown) and chloroplasts were removed. Data were normalized using variance stabilizing transformation (vds/vst) in R with the package DESeq2 (Love, Huber, & Anders, 2014; R Core Team, 2018). Although the Silva reference data set v. 132 considers Betaproteobacteria within Gammaproteobacteria (as Betaproteobacteriales order), in this study, we still refer to them as Betaproteobacteria class.

For the downstream analysis, 97 (3,919,437 total sequences) out of 101 samples were included. In GKS (*n* = 64), 2,368 ASVs were assigned (range: 75 to 237, mean = 136.7, *SD* = 42.6) corresponding to 2,371,284 sequences. In PIB (*n* = 33), we assigned 2,206 ASVs (range: 168 to 499, mean = 332.6, *SD* = 117.6) with 1,547,908 sequences.

### 2.5 Diversity and statistical analyses

The asymptotic estimates of species richness, Shannon and Simpson diversity indexes were calculated using the iNEXT package in R (Hsieh, Ma, & Chao, 2016; R Core Team, 2018), which provides simple functions to compute and to plot the seamless rarefaction and extrapolation sampling curves of the Hill numbers. Further, the phylogenetic tree used for Faith's PD (Faith, 1992) was calculated using FastTree v. 2.1.7, applying the generalized time‐reversible model. Bray–Curtis similarity (transformed to percentages and based on the relative abundance of ASVs), ordinations (metaMDS), fit of environmental vectors into ordinations and statistical differences between lakes (ANOSIM) were calculated in the Vegan package in R (R Oksanen et al., 2019; Core Team, 2018). The analysis of multivariate homogeneity of group dispersion (variance) was done using the betadisper function implemented in the Vegan package (Oksanen et al., 2019). A permutation analysis (*n* = 999) was done to test for significance using the permutest function in the same package (Oksanen et al., 2019).

Taxonomically distinctive members between lakes were detected using a linear discriminant analysis (LDA) effective size (LEfSe) in the galaxy server (http://huttenhower.sph.harvard.edu/galaxy/) (Segata et al., 2011). Briefly, a Kruskal–Wallis test analysis (alpha = 0.05) was conducted to test whether the values in different classes were differentially distributed. Then, a pairwise Wilcoxon test (alpha = 0.05) was used to check whether all pairwise comparisons between subclasses within different classes significantly agree with the class level. Finally, the results were used to build a linear discriminant analysis model (threshold = 2.0) from which the relative difference among classes is used to rank the taxonomically distinctive members.

### 2.6 Stochastic and deterministic assessment

To evaluate the relative influence of stochastic and deterministic processes, and to identify which system features impose selection to bacterial communities in the studied lakes, we used the analytical framework proposed by Stegen et al. (2013). This framework relies on phylogenetic turnover and therefore requires testing for a phylogenetic signal which shows that more closely related taxa have more similar habitat associations (Stegen et al., 2012). First, we found the set of environmental parameters that in combination correlates strongest with the overall change in community composition of both lakes (Andersson, Riemann, & Bertilsson, 2010). This was done using the BIOENV function in the Vegan package in R (R Oksanen et al., 2019; Core Team, 2018). In GKS, the highest correlation was obtained with a combination of water temperature and pH (*Spearman's correlation coefficient* = 0.6), while in PIB it was a combination of water temperature and oxygen (*Spearman's correlation coefficient* = 0.3240). Then, we calculated the abundance‐weighted mean of the selected environmental parameters (water temperature and pH for GKS; water temperature and oxygen for PIB) for each ASV. For example, we extracted from all records for a given ASV the water temperature and the ASV abundance, and then computed the abundance‐weighted mean water temperature (Stegen et al., 2012). Finally, the phylogenetic signal was tested with the abundance‐weighted selected parameters and the phylogenetic tree using the phylocorrelogram function in the package phylosignal (Keck, Rimet, Bouchez, & Franc, 2016) (Figure S1).

Phylogenetic beta diversity was quantified using the mean nearest taxon distance (βMNTD) using the function ‘comdistnt’ (abundance.weighted = true) from the package ‘picante’ (Kembel et al., 2010). Null models in which the tips of the phylogenetic tree are randomized (*n* = 999) were applied to calculate the null βMNTD. Then, we calculated the β‐nearest taxon index (βNTI), which is the difference between the observed βMNTD and the mean of the null distribution of βMNTD normalized by its standard deviation. We compared βMNTD and βNTI across all possible pairwise combinations. βNTI values >+2 of pairwise comparison indicate variable selection (i.e. high compositional turnover primarily caused by a shift in environmental factors), and values <−2 indicate homogeneous selection (consistent environmental factors primarily cause low compositional turnover). If the pairwise comparison shows |βNTI| values < 2, then it indicates that stochastic processes drive the observed difference in phylogenetic community composition (Stegen et al., 2013). Therefore, to determine the relative contribution of stochastic processes, we calculated the Raup‐Crick metric based on Bray–Curtis (RCbray). Pairwise comparisons with RCbray>+0.95 and |βNTI| < 2 correspond to dispersal limitation (high turnover in composition is mainly caused by a low rate of dispersal enabling community composition to drift apart). RCbray <−0.95 and |βNTI| < 2 correspond to homogenizing dispersal, which is similar to mass effects and source‐sink dynamics; however, these terms invoke additional assumptions and processes (Stegen et al., 2013). Therefore, homogenizing dispersal simply indicates that dispersal is high enough to cause low turnover by overwhelming other processes. Pairwise comparisons with |RCbray| < 0.95 correspond to drift (undominated processes) (Stegen et al., 2015).

The identification of system features that impose selection was done computing the distance‐based Moran's eigenvector maps (function dbMEM) within package “adespatial” (Dray et al., 2019; R Core Team, 2018). Then, we combined the temporal eigenvectors with measured abiotic parameters (water temperature, electrical conductivity, DOC, DN, pH, and ice presence; oxygen was included only in PIB) using principal components analysis (PCA). The obtained PCA axes were used as independent variables in a model‐selection procedure with βNTI (normalized to vary between 0 and 1) as the dependent variable using the function capscale and ordiR2step within package “vegan” in R (Oksanen et al., 2019; R Core Team, 2018). When a given PCA axis is significant for βNTI, but the measured abiotic variables do not load onto it, the PCA axis is considered to represent an unmeasured environmental variable that imposes selection (Stegen et al., 2013). By contrast, when a measured abiotic variables load heavily onto a significant PCA axis, the axis is considered to be a measured environmental variable that imposes selection (Stegen et al., 2013).

## 3 RESULTS

### 3.1 Environmental data

Values for most water physicochemical parameters and chlorophyll‐*a* were significantly higher in PIB than in GKS except for pH and nitrate, which were significantly higher in GKS (*p* < 0.05; Figure S2). The mean lowest temperature for the water column in GKS ranged between 0.2°C and 2.6°C (mean: 1°C; February 2016) and ranged between 2.3°C and 4.5°C in PIB (mean: 3.4°C; February 2015). The mean maximum temperature for the water column in GKS ranges between 7.2°C and 17.3°C (mean: 13.4°C; July 2015); and in PIB ranged between 3.2°C and 17.6°C (mean: 10.4°C; September 2015) (Figure S3). In GKS, an increase in DN was found during the ice‐covered period with a peak in April 2015 (Figure S3a). At the short‐time scale, GKS showed large differences in water temperature, conductivity and chlorophyll‐*a* in the water column as indicated by the high standard deviation (Figure S3b). In PIB, water temperature, DOC and oxygen concentrations also showed a large standard deviation in the water column during the ice‐free period (Figure S3c).

### 3.2 Diversity metrics

**Figure 1:**
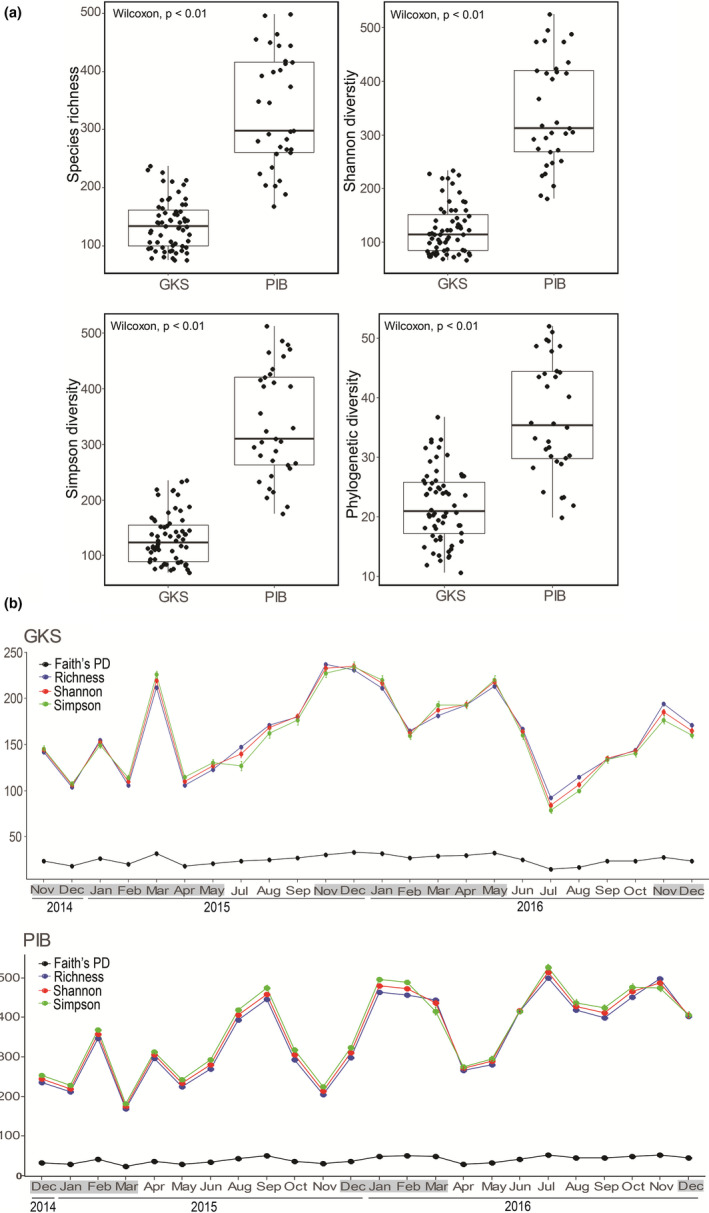
(a) Asymptotic estimates of species richness, Shannon diversity, Simpson diversity and phylogenetic diversity (Faith's PD) for the bacterial community from Gossenköllesee (GKS) and Piburgersee (PIB). (b) changes in diversity estimates for GKS and PIB during the annual scale. Months in grey indicate the ice‐covered period [Colour figure can be viewed at wileyonlinelibrary.com]

Alpha diversity metrics were significantly higher in PIB than in GKS (Wilcoxon test, *p* < .01; Figure 1a). Alpha diversity in GKS was highest during the ice‐covered period (richness_mean_ = 172, Shannon_mean_ = 173, Simpson_mean_ = 173, PD_mean_ = 26) than during the ice‐free one (richness_mean_ = 144, Shannon_mean_ = 140, Simpson_mean_ = 135, PD_mean_ = 23) (Figure 1b). In PIB, the same metrics reached the highest mean values during 2016 (Figure 1b). At the short‐term sampling, diversity metrics in GKS were more stable than at the annual one (Figure S4a). In the water column, the diversity metrics showed the highest values at 0, 6 and 21 m in PIB and at 8 m depth in GKS (Figure S4b).

**Figure 2:**
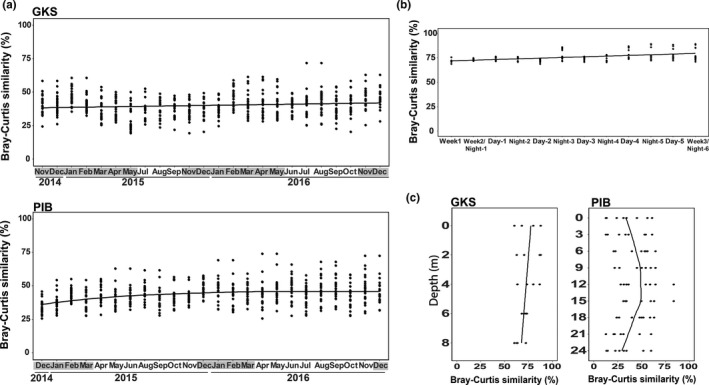
Bray–Curtis similarity of bacterial communities between November 2014 and December 2016 in Gossenköllesee (GKS) and between December 2014 and December 2016 in Piburgersee (PIB) (a), during the short‐term sampling in GKS (b) and for the water column (c) of both lakes. Seasonal trends (black lines) are shown. Months in grey indicate the ice‐covered period [Colour figure can be viewed at wileyonlinelibrary.com]

The bacterial community of both lakes showed similar values of Bray–Curtis similarity (range: 19.4%‐73.8%; Figure 2a). GKS was the only lake showing a seasonal pattern with slightly higher similarity values observed during the ice‐covered period. In GKS, Bray–Curtis similarity values were higher at the short‐term sampling scale than at the annual one (Figure 2b). Bray–Curtis similarity calculated for the water column in GKS showed that communities at the surface (upper 0.5 m) and 2 m depth were more similar (Figure 2c), whereas in PIB, communities were more similar between 9 m and 18 m in the hypolimnion.

### 3.3 Bacterial community composition

**Figure 3:**
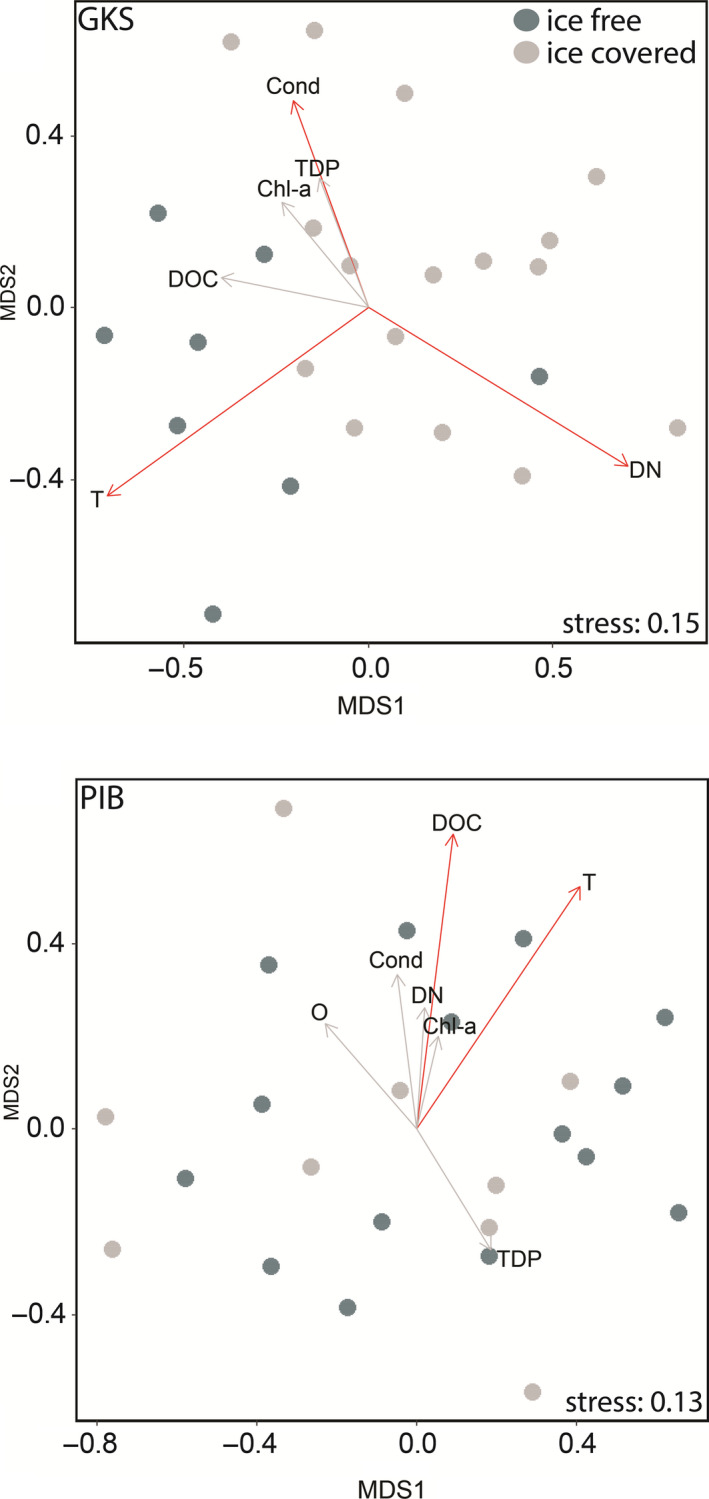
Nonmetric Multidimensional Scaling (NMDS) analysis based on Bray–Curtis dissimilarity between the ice‐covered and ice‐free period in Gossenköllesee (GKS) and Piburgersee (PIB), showing the environmental parameters (significant parameters are shown with red arrows). Chl‐α, chlorophyll‐α. Cond, electrical conductivity. DN, dissolved nitrogen. DOC, dissolved organic carbon. O, dissolved oxygen. T, water temperature. TDP, total dissolved phosphorus [Colour figure can be viewed at wileyonlinelibrary.com]

The community composition was significantly different between the ice‐covered and ice‐free periods in both lakes (ANOSIM for GKS, R: 0.41, *p* < .001; ANOSIM for PIB, R: 0.18, *p* < .021). However, ordination analyses, based on Bray–Curtis dissimilarity, showed that the bacterial community in GKS was more segregated according to periods than in PIB (ice‐free versus ice‐covered; Figure 3). DN concentration (*R* ^2^ = 0.63), water temperature (*R* ^2^ = 0.69) and electrical conductivity (*R* ^2^ = 0.27) were significant variables explaining the segregation in communities from GKS (*p* < .05), whereas in PIB, water temperature (*R* ^2^ = 0.44) and DOC concentration (*R* ^2^ = 0.41) were significant (*p* < .01). The multivariate homogeneity test analysis, betadisper, showed a lower dispersion around the median in GKS than in PIB (distance to centroids, GKS = 0.3377, PIB = 0.4196), and a significant difference between lakes, according to the permutation test (*p* < .01).

**Figure 4:**
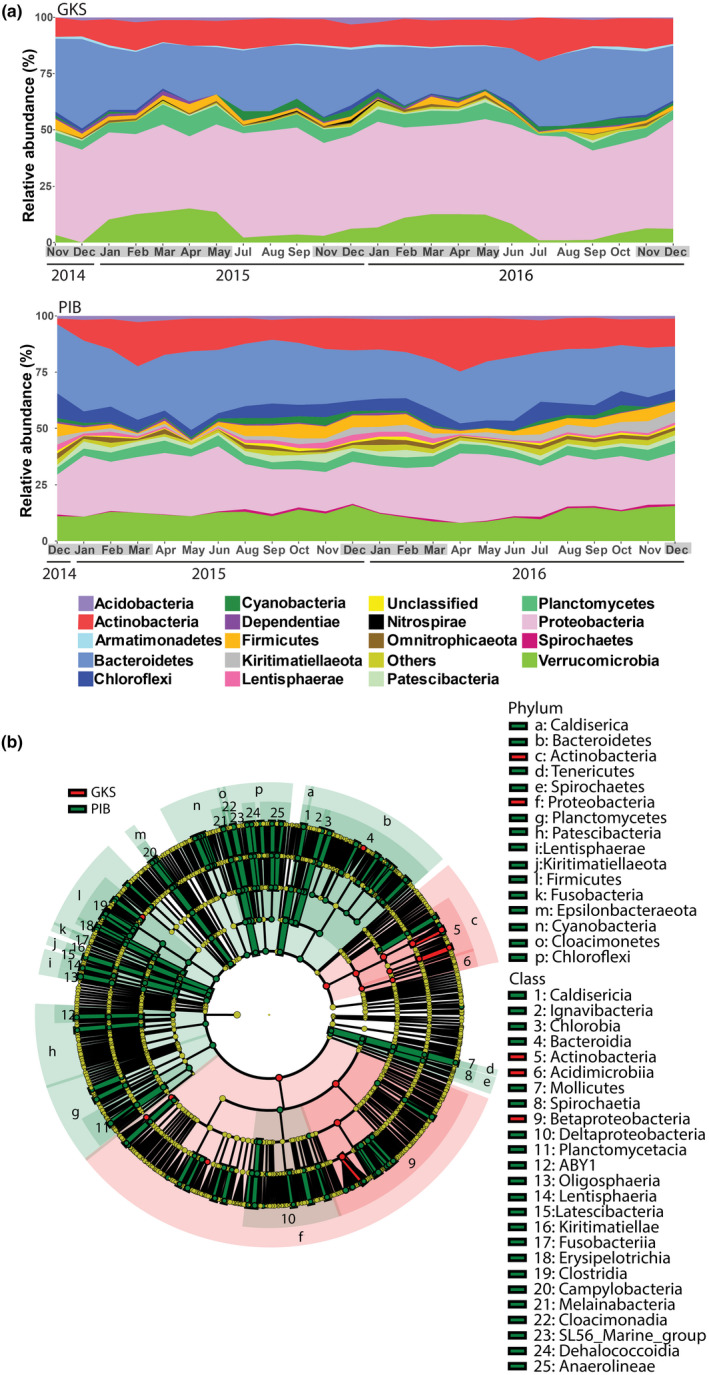
(a) Temporal changes in bacterial relative abundance in Gossenköllesee (GKS) and in Piburgersee (PIB). (b) The cladogram visualizes the output of the LEfSe algorithm, which identifies significant taxonomical differences between GKS and PIB [Colour figure can be viewed at wileyonlinelibrary.com]

In both lakes, the main abundant phyla at the annual scale were Actinobacteria, Bacteroidetes, Proteobacteria, and Verrucomicrobia (Figure 4a). In GKS, 14 out of 27 phyla showed a relative abundance >1%, at least in one month. Proteobacteria was the most abundant phylum, up to 48.5% of relative abundance, with Betaproteobacteria (1.1%–30%) and Alphaproteobacteria (12.7%–27.7%) as the most abundant classes. Verrucomicrobia showed a high relative abundance during the ice‐covered period (6.6%–13.4%; Figure 4a) and a low relative abundance during the ice‐free period (0.8%–3.4%; Figure S5a). The number of phyla in PIB was higher than in GKS, but only 16 out of 38 were present with relative abundances >1%. Actinobacteria was the only phylum showing an increase in relative abundance at the end of the ice‐covered period in PIB (March 2015 and April 2016). In the water column of GKS, the same relative abundance of phyla was found (Figure S5b). By contrast, in the water column of PIB a higher number of phyla were found from 18 m depth (Figure S5b). The most abundant genera did not show a clear seasonality in GKS and PIB (Figure S6), except for those within Verrucomicrobia in the former. The most abundant genera in GKS were the Hgcl clade and unknown genera within Proteobacteria in both periods, and unknown genera within Verrucomicrobia only observed during the ice‐covered period. In PIB, the most abundant genera were Hgcl clade (Actinobacteria) and unknown genera from Bacteroidetes, Proteobacteria and Verrucomicrobia. During the short‐term sampling in GKS, members of the bacterial community with high relative abundance were the Hgcl clade and unknown genera within Bacteroidetes and Proteobacteria.

A total of 230 significant taxonomical differences were identified between GKS and PIB (Table S1). Actinobacteria (Acidimicrobiia and Actinobacteria) and Proteobacteria (Betaproteobacteria) were the only phyla identified with significantly higher abundance in GKS than in PIB (Kruskal–Wallis test, *p* < .05) (Figure 4b). Instead, 14 phyla were identified as significant members of PIB (Kruskal–Wallis test, *p* < .05; Figure 4b; Table S1).

### 3.4 Community assembly processes and relevant variables that impose them

**Figure 5:**
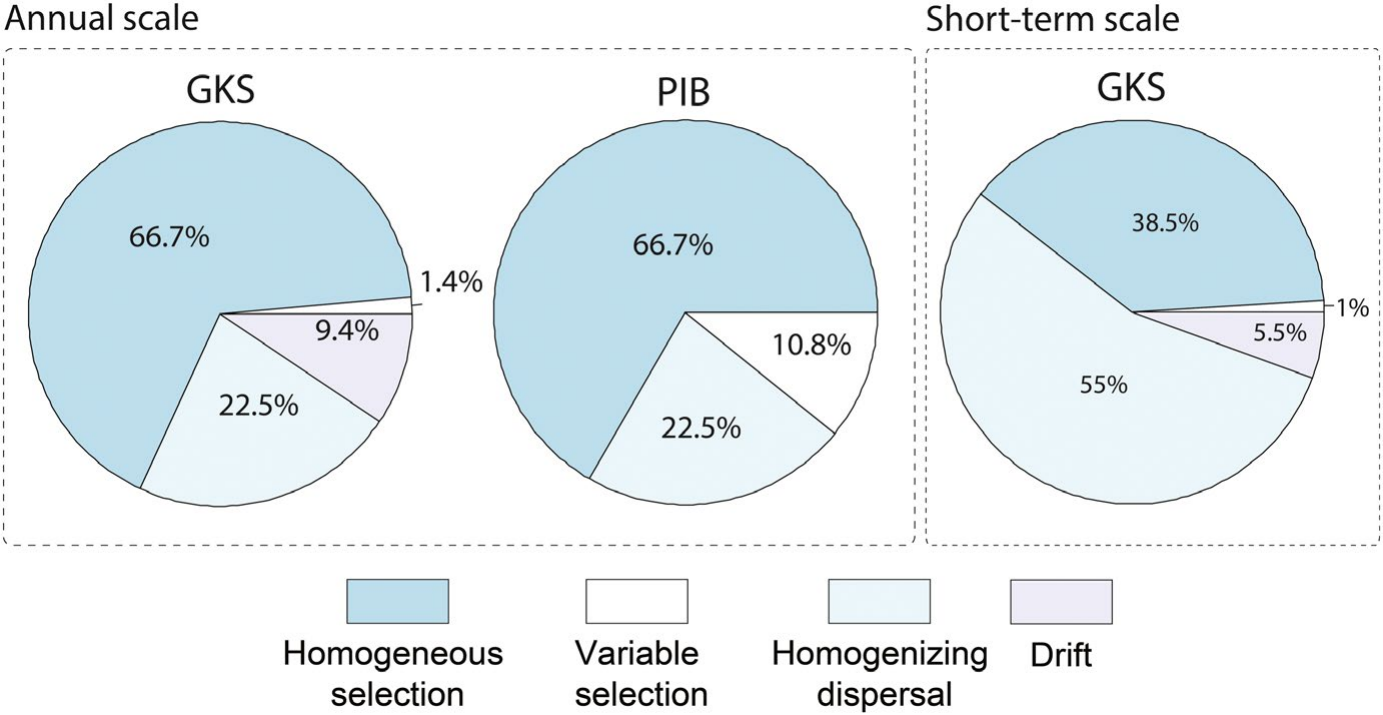
Contribution of different ecological processes to the assembly of the bacterial community in Gossenköllesee (GKS) and in Piburgersee (PIB) at the annual scale and at short‐term scale in GKS [Colour figure can be viewed at wileyonlinelibrary.com]

The same predominant assembly processes were found in both lakes during two consecutive years (Figure 5). Homogeneous selection, which explained 66.7% of community turnover, was the most important ecological process, followed by homogenizing dispersal, which explained 22.5% of community turnover. Drift had a low influence (9.4%) in GKS, and variable selection was more important in PIB than in GKS (GKS: 1.4%; PIB: 10.8%). In contrast, at the short‐term, the main ecological process structuring the bacterial community in GKS was homogenizing dispersal (55%) followed by homogeneous selection (38.5%).

The analysis of ecosystem variables that impose selection in GKS showed five significant PCA axes for βNTI (PC2, PC14, PC19, PC22 and PC23; Table S2). Water temperature, electrical conductivity, DOC, DN, and ice cover were weakly loaded onto PC2, but pH was the strongest variable loading onto this axis. There were no measured environmental variables loaded on PC14, PC19, PC22, and PC23. In PIB, PC9 was the only significant for βNTI, with no environmental variables loaded onto this axis (Table S3).

## 4 DISCUSSION

Assessing the relative contribution of deterministic and stochastic processes structuring communities over temporal scales is a primary step to understand how they will respond to local, regional and global changes (Shade et al., 2007). However, proper quantification of these processes for microbial communities has been only possible recently (Liu et al., 2020; Logares et al., 2020; Stegen et al., 2013; Vass et al., 2020; Yan et al., 2017). For example, studies in lakes found that deterministic processes govern the structure of bacterial communities at different temporal scales (Kent, Yannarell, Rusak, Triplett, & McMahon, 2007; Wang et al., 2013). By contrast, stochastic processes seem to explain the spatial structure of lake bacterial communities at local and regional scales (Roguet et al., 2015). Here we show that deterministic processes, mainly homogeneous selection, primarily governed the bacterial community structure of two mountain lakes over an annual scale, but that homogenizing dispersal, a stochastic process, structured the bacterioplankton composition in the alpine lake during short‐term sampling.

Several studies have shown that bacterial communities have recurrent or predictable temporal variability (Li et al., 2015; Salmaso et al., 2018; Shade et al., 2007; Yannarell & Triplett, 2005), but this is not always observed (Kent et al., 2004; Linz et al., 2017; Tammert et al., 2015). In fact, seasonal patterns in bacterial community composition have been found to vary depending on the year and lake considered (Jones, Cadkin, Newton, & McMahon, 2012; Rusak, Jones, Kent, Shade, & McMahon, 2009). Our hypothesis that temporal variability differs across various time scales and trophic states was validated, as we found temporal differences in diversity and composition between the lakes and time scales (long‐ versus. short‐term in GKS). Further, community segregation between the ice‐free and ice‐covered periods was only found in the alpine oligotrophic lake and was driven by changes in environmental filters such as water temperature and nutrient concentration (e.g., dissolved nitrogen). In contrast, recurrent seasonal changes were not observed in the mesotrophic lake (PIB) at the taxonomical level and time scale considered. The absence of seasonal changes in PIB, however, could have been masked by the sampling strategy used here (i.e., composite samples), because it has been described that there are differences in bacterial temporal variability and composition between the epilimnion and hypolimnion of stratified lakes (Shade, Jones, & McMahon, 2008).

At the annual scale, we found the same relative contribution of homogeneous selection and homogenizing dispersal, despite the clear differences in environmental conditions, alpha diversity and bacterial seasonal variability between the two studied lakes. This supports our hypothesis regarding changes in ecological processes at different time scales.

When homogeneous selection is identified in a time‐series data set, we infer that the low compositional turnover is caused by environmental factors that are relatively predictable over time (e.g., high water temperatures every summer and low ones every winter). In contrast, variable selection would take place when environmental factors change with irregular temporal patterns such as those caused by a storm event, producing a high compositional turnover. When stochastic processes are detected, a high rate of dispersal and a low turnover in composition (homogenizing dispersal) is expected when the same habitat is sampled throughout time. For example, when we observe the presence of homogenizing dispersal in a pairwise comparison of two samples from a few months apart, we infer that the main bacterial community composition is kept through the elapsed time. Instead, dispersal limitation is expected to be high, when two different habitats (or samples) are not connected. The absence in our data set of dispersal limitation through the year (and between months) suggests that the bacterial community within a lake does not completely change; however, this could be influenced by the composite sampling strategy used here.

Selection‐related processes have been reported to be dominant in lakes on the long‐term (Yan et al., 2017), whereas dispersal‐related processes seem to dominate in small aquatic ecosystems on the short‐term (Vass et al., 2020). Although more quantitative analyses are necessary in different types of lakes to reach a generalization, in a study of subsurface microbial communities, Stegen et al. (2012) suggested that deterministic and stochastic processes are guided by general rules across ecosystems. We propose that lake bacterioplankton are governed by a balance of deterministic and stochastic processes, with the former being most relevant at long‐term scales, and the latter at short‐term scales. We further argue that the recurrent changes in bacterial composition over a long‐term scale produced by environmental filters (i.e. biotic and abiotic) are the most important processes shaping communities, but the impact of these environmental filters decreases at short‐term scales, opening the possibility for stochastic ones (e.g. homogenizing dispersal) to become more important. Identifying those environmental filters is challenging and largely depends on the battery of parameters included. According to the model‐selection procedure, all tested variables in GKS (i.e., water temperature, conductivity, DOC, DN, ice presence, and pH) appear to impose selection, while in PIB, it remains unclear which are the main environmental filters imposing selection.

The quantification of ecological processes responsible for community assembly using the Stegen framework has limitations. For example, this framework does not parse out sub‐classes of selection, such as competition and trophic interactions, and it could be sensitive to factors such as phylogenetic uncertainty and alpha diversity underestimation (Stegen et al., 2013). Further, the selection‐related processes could include deterministic components of dispersal (e.g., active propulsion) and some degree of diversification, such as those derived from favourable mutations (Zhou & Ning, 2017). Further, homogenizing dispersal is similar to mass effect and source‐sink dynamics (Stegen et al., 2013), and dispersal limitation may depict processes such as historical contingency, phylogenetically nonconserved selection and other unmeasured processes (Vass et al., 2020). Therefore, the outcomes from this framework do not represent a definitive explanation of assembly processes occurring at a specific temporal scale, since not all of the ecological processes are being quantified. However, this method is a first approach to compare major ecological processes (i.e. variable selection, homogeneous selection, dispersal limitation, homogenizing dispersal and undominated processes) through space and time.

Another potential bias using this framework could rely on sampling and data analysis strategies. For example, the use of different approaches to studying the same temporal scale (e.g., replicate versus single samples), different reads cluster strategy (e.g., OTUs versus ASVs) and the use of relative or absolute abundance data could lead to different conclusions about the balance of ecological processes. Therefore, the use of the same methodology/sampling strategy among lakes is needed to reach consistent general patterns on dominant assembly processes.

The temporal variability in bacterial community changed across different time scales and trophic state. Overall, bacterial community variability from the oligotrophic lake was more stable through the two consecutive years and less dispersed compared with that of the mesotrophic one. Verrucomicrobia was the only phylum showing a clear, repeated seasonality in GKS with high abundance during the ice‐covered period (average relative abundance of 9.3%) and a low one during the ice‐free period (average relative abundance of 2.9%). By contrast, this phylum showed a stable relative abundance (ca. 12%) in PIB for the whole year. The prevalence of this phylum during winter seems to be typical for temperate and boreal lakes (Tran, Ramachandran, & Khawasik, 2018). Nevertheless, the detection of Verrucomicrobia in freshwaters has been compromised by methodological issues (e.g., primers bias (McCarthy, Chiang, Schmidt, & Denef, 2015)), and therefore, there are contrasting reports regarding its numerical importance or dominance (Chiang et al., 2018; Llames, Huber, Metz, & Unrein, 2017; Newton, Jones, Eiler, McMahon, & Bertilsson, 2011). Our results additionally suggest that differences in the relative abundance of this phylum lie in its seasonality and in the trophic state of the lake considered.

Betaproteobacteria and Alphaproteobacteria are usually the numerically most important groups in GKS (Alfreider et al., 1996; Pernthaler et al., 1998). We found *Limnohabitans* spp. and *Rhodoferax* spp. to be important components within Betaproteobacteria (despite the high abundance of unknown genera within this class). The first genus did not show a clear seasonality, but the highest relative abundance was found during the ice‐free period (July 2016). Instead, *Rhodoferax* spp. showed the highest relative abundance in the last months of the ice‐covered period (April–May). *Limnohabitans* spp. and *Rhodoferax* spp. are aerobic anoxygenic phototrophs (AAPs) and dominate the AAP communities in many lakes (Ruiz‐González, Garcia‐Chaves, Ferrera, Niño‐García, & Del Giorgio, 2020; Salka, Cuperová, Mašín, Koblížek, & Grossart, 2011). However, *Rhodoferax* spp. has not been described as an abundant AAP species in GKS. Actually, the main AAP species previously found in GKS during the ice‐free season were related to *Sandarakinorhabdus limnophila*, *Erythromonas ursincola* and *Sphingomonas* (Čuperová, Holzer, Salka, Sommaruga, & Koblízek, 2013). Besides the high abundance of *Rhodoferax* spp., we also found that *Sandarakinorhabdus* spp. and *Sphingomonas* spp. were the main representatives of the AAP community, but *Erythromonas* spp. was found in a low relative abundance. Further, we showed that AAP species exhibit high abundance not only during the ice‐free period, but also during the ice‐covered period. Another bacterial group that accounts for >50% of the total bacterial biomass after ice break‐up in GKS is Bacteroidetes (Wille, Sonntag, Sattler, & Psenner, 1999). This group is also abundant in lakes located in different climatic zones (Schauer & Hahn, 2005). Some organisms within this group in oligotrophic lakes are related to *Haliscomenobacter* species (Hahn & Schauer, 2007). We detected the taxa *Haliscomenobacter* with a relative abundance >1% only in GKS, and its abundance increased during the ice‐break period and persisted during the whole ice‐free period.

Actinobacteria, Bacteroidetes and Betaproteobacteria are the most important groups in PIB. Actinobacteria is numerically important in the oxygenated water layers (epilimnion), and the other two are more abundant in the oxygen‐depleted hypolimnion (Salcher, Pernthaler, & Posch, 2010). The same groups were found in our study; however, we highlight the high relative abundance of Verrucomicrobia, which has not been previously described.

In general, the ice‐free months are the most studied periods in lakes based on the notion that the winter was considered a “dormant season” (Campbell, Mitchell, Groffman, Christenson, & Hardy, 2005; Hampton et al., 2017). Most of the bacterial diversity patterns, their ecological role and potential metabolic pathways in lakes in general, as well as in GKS and PIB, have been derived from the ice‐free period (Hörtnagl, Pérez, Zeder, & Sommaruga, 2010; Pérez & Sommaruga, 2011; Wille et al., 1999). However, the ice‐covered period is crucial and influences the structure of the bacterial community and thus, many key ecological processes (Bertilsson et al., 2013; Grosbois, Mariash, Schneider, & Rautio, 2017; Hampton et al., 2017). For example, potential bacterial metabolic activities such as sulphur and methane oxidation have been proposed as important processes during this period (Bertilsson et al., 2013; Schütte, Cadieux, Hemmerich, Pratt, & White, 2016). The duration of the ice cover is longer in GKS than in PIB and probably explains why bacterioplankton segregation was clearer compared with the ice‐free period. Further, the bacterioplankton in GKS showed the highest diversity metrics during the ice‐cover season, which may be influenced by the different habitats generated in the ice cover and its interaction with the water column (Felip, Wille, Sattler, & Psenner, 2002). Instead, the ice‐cover period of PIB did not change diversity metrics during the two years. The only change observed in PIB during the ice‐cover season was the increase in the relative abundance of the Hgcl clade between the last months of this season and the ice‐break period.

## 5 CONCLUSIONS

Assessing bacterioplankton variability at different temporal scales is important to elucidate which taxonomical group is relevant to key environmental processes and biogeochemical cycles. Here, we showed that annual changes in bacterioplankton of both lakes were primarily controlled by the same relative contribution of a deterministic process (homogeneous selection), although alpha diversity and patterns of bacterial variability changed according to the trophic state of the lake. The dominance of deterministic processes changed when the bacterioplankton variability was assessed at a shorter time scale, and in this case, a stochastic process (homogenizing dispersal) was the most important. Thus, we conclude that deterministic processes will influence the bacterioplankton equally across lakes at long‐term scales, whereas stochastic processes play a secondary role, regardless of bacterioplankton composition or trophic state of the lake.

## AUTHORS' CONTRIBUTION

P.A. collected (in part) the samples, prepared the samples for Illumina sequencing and run the bioinformatics analysis, P.A. and R.S. wrote the manuscript, and R.S. obtained funding for the project. Both authors have read and approved this manuscript.

## Supporting information

Supplementary Material

## Data Availability

The data sets generated and/or analysed during the current study are available in the Sequence Read Archive (SRA) of NCBI repository, under Accession no SRP167155.

## References

[mec15538-bib-0001] Alfreider, A. , Pernthaler, J. , Amann, R. , Sattler, B. , Glockner, F. , Wille, A. , & Psenner, R. (1996). Community analysis of the bacterial assemblages in the winter cover and pelagic layers of a high mountain lake by in situ hybridization. Applied and Environmental Microbiology, 62(6), 2138–2144. 10.1128/AEM.62.6.2138-2144.1996 16535341 PMC1388879

[mec15538-bib-0002] Andersson, A. F. , Riemann, L. , & Bertilsson, S. (2010). Pyrosequencing reveals contrasting seasonal dynamics of taxa within Baltic Sea bacterioplankton communities. The ISME Journal, 4(2), 171–181. 10.1038/ismej.2009.108 19829318

[mec15538-bib-0003] Bertilsson, S. , Burgin, A. , Carey, C. C. , Fey, S. B. , Grossart, H.‐P. , Grubisic, L. M. , … Smyth, R. L. (2013). The under‐ice microbiome of seasonally frozen lakes. Limnology and Oceanography, 58(6), 1998–2012. 10.4319/lo.2013.58.6.1998

[mec15538-bib-0004] Callahan, B. J. , McMurdie, P. J. , & Holmes, S. P. (2017). Exact sequence variants should replace operational taxonomic units in marker‐gene data analysis. The ISME Journal, 11, 2639–2643. 10.1038/ismej.2017.119 28731476 PMC5702726

[mec15538-bib-0005] Callahan, B. J. , McMurdie, P. J. , Rosen, M. J. , Han, A. W. , Johnson, A. J. A. , & Holmes, S. P. (2016). DADA2: High‐resolution sample inference from Illumina amplicon data. Nature Methods, 13, 581–583. 10.1038/nmeth.3869 27214047 PMC4927377

[mec15538-bib-0006] Campbell, J. L. , Mitchell, M. J. , Groffman, P. M. , Christenson, L. M. , & Hardy, J. P. (2005). Winter in northeastern North America: A critical period for ecological processes. Frontiers in Ecology and the Environment, 3(6), 314–322. 10.1890/1540-9295(2005)003[0314:WINNAA]2.0.CO;2

[mec15538-bib-0007] Caruso, T. , Chan, Y. , Lacap, D. C. , Lau, M. C. Y. , McKay, C. P. , & Pointing, S. B. (2011). Stochastic and deterministic processes interact in the assembly of desert microbial communities on a global scale. The ISME Journal, 5, 1406–1413. 10.1038/ismej.2011.21 21368908 PMC3160679

[mec15538-bib-0008] Chase, J. M. (2010). Stochastic community assembly causes higher biodiversity in more productive environments. Science, 328(5984), 1388–1391. 10.1126/science.1187820 20508088

[mec15538-bib-0009] Chase, J. M. , & Myers, J. A. (2011). Disentangling the importance of ecological niches from stochastic processes across scales. Philosophical Transactions of the Royal Society B: Biological Sciences, 366, 2351–2363. 10.1098/rstb.2011.0063 PMC3130433 21768151

[mec15538-bib-0010] Chen, W. , Ren, K. , Isabwe, A. , Chen, H. , Liu, M. , & Yang, J. (2019). Stochastic processes shape microeukaryotic community assembly in a subtropical river across wet and dry seasons. Microbiome, 7, 138 10.1186/s40168-019-0749-8 31640783 PMC6806580

[mec15538-bib-0011] Chiang, E. , Schmidt, M. L. , Berry, M. A. , Biddanda, B. A. , Burtner, A. , Johengen, T. H. , … Denef, V. J. (2018). Verrucomicrobia are prevalent in north‐temperate freshwater lakes and display class‐level preferences between lake habitats. PLoS One, 13, e0195112 10.1371/journal.pone.0195112 29590198 PMC5874073

[mec15538-bib-0012] Čuperová, Z. , Holzer, E. , Salka, I. , Sommaruga, R. , & Koblízek, M. (2013). Temporal changes and altitudinal distribution of aerobic anoxygenic phototrophs in mountain lakes. Applied and Environmental Microbiology, 79(20), 6439–6446. 10.1128/AEM.01526-13 23956384 PMC3811222

[mec15538-bib-0013] Dai, W. , Zhang, J. , Tu, Q. , Deng, Y. E. , Qiu, Q. , & Xiong, J. (2017). Bacterioplankton assembly and interspecies interaction indicating increasing coastal eutrophication. Chemosphere, 177, 317–325. 10.1016/j.chemosphere.2017.03.034 28319885

[mec15538-bib-0014] Dray, B. , Bauman, D. , Blanchet, G. , Borcard, D. , Clappe, S. , Guenard, G. , … Wagner, H. H. (2019). adespatial: Multivariate multiscale spatial analysis. R package.

[mec15538-bib-0015] Faith, D. P. (1992). Conservation evaluation and phylogenetic diversity. Biological Conservation, 61, 1–10. 10.1016/0006-3207(92)91201-3

[mec15538-bib-0016] Felip, M. , Wille, A. , Sattler, B. , & Psenner, R. (2002). Microbial communities in the winter cover and the water column of an alpine lake: System connectivity and uncoupling. Aquatic Microbial Ecology, 29, 123–134. 10.3354/ame029123

[mec15538-bib-0017] Fuhrman, J. A. , Cram, J. A. , & Needham, D. M. (2015). Marine microbial community dynamics and their ecological interpretation. Nature Reviews. Microbiology, 13(3), 133–146. 10.1038/nrmicro3417 25659323

[mec15538-bib-0018] Grosbois, G. , Mariash, H. , Schneider, T. , & Rautio, M. (2017). Under‐ice availability of phytoplankton lipids is key to freshwater zooplankton winter survival. Scientific Reports, 7(1), 11543 10.1038/s41598-017-10956-0 28912552 PMC5599675

[mec15538-bib-0019] Hahn, M. W. , & Schauer, M. (2007). “Candidatus Aquirestis calciphila” and “Candidatus Haliscomenobacter calcifugiens”, filamentous, planktonic bacteria inhabiting natural lakes. International Journal of Systematic and Evolutionary Microbiology, 57, 936–940. 10.1099/ijs.0.64807-0 17473236

[mec15538-bib-0020] Hampton, S. E. , Galloway, A. W. E. , Powers, S. M. , Ozersky, T. , Woo, K. H. , Batt, R. D. , … Xenopoulos, M. A. (2017). Ecology under lake ice. Ecology Letters, 20, 98–111. 10.1111/ele.12699 27889953

[mec15538-bib-0021] Hörtnagl, P. , Pérez, M. T. , Zeder, M. , & Sommaruga, R. (2010). The bacterial community composition of the surface microlayer in a high mountain lake. FEMS Microbiology Ecology, 73(3), 458–467. 10.1111/j.1574-6941.2010.00904.x 20528985 PMC2955963

[mec15538-bib-0022] Hsieh, T. C. , Ma, K. H. , & Chao, A. (2016). iNEXT: An R package for rarefaction and extrapolation of species diversity (Hill numbers). Methods in Ecology and Evolution / British Ecological Society, 7(12), 1541–1546. 10.1111/2041-210X.12613

[mec15538-bib-0081] Jia, X. , Dini‐Andreote, F. , & Falcão Salles, J. (2018). Community Assembly Processes of the Microbial Rare Biosphere. Trends in Microbiology, 26(9), 738–747. 10.1016/j.tim.2018.02.011 29550356

[mec15538-bib-0023] Jones, S. E. , Cadkin, T. A. , Newton, R. J. , & McMahon, K. D. (2012). Spatial and temporal scales of aquatic bacterial beta diversity. Frontiers in Microbiology, 3, 318 10.3389/fmicb.2012.00318 22969757 PMC3431545

[mec15538-bib-0024] Keck, F. , Rimet, F. , Bouchez, A. , & Franc, A. (2016). phylosignal: An R package to measure, test, and explore the phylogenetic signal. Ecology and Evolution, 6(9), 2774–2780. 10.1002/ece3.2051 27066252 PMC4799788

[mec15538-bib-0025] Kembel, S. W. , Cowan, P. D. , Helmus, M. R. , Cornwell, W. K. , Morlon, H. , Ackerly, D. D. , … Webb, C. O. (2010). Picante: R tools for integrating phylogenies and ecology. Bioinformatics, 26(11), 1463–1464. 10.1093/bioinformatics/btq166 20395285

[mec15538-bib-0026] Kent, A. D. , Jones, S. E. , Yannarell, A. C. , Graham, J. M. , Lauster, G. H. , Kratz, T. K. , & Triplett, E. W. (2004). Annual patterns in bacterioplankton community variability in a humic lake. Microbial Ecology, 48(4), 550–560. 10.1007/s00248-004-0244-y 15696388

[mec15538-bib-0027] Kent, A. D. , Yannarell, A. C. , Rusak, J. A. , Triplett, E. W. , & McMahon, K. D. (2007). Synchrony in aquatic microbial community dynamics. The ISME Journal, 1, 38–47. 10.1038/ismej.2007.6 18043612

[mec15538-bib-0080] Ladau, J. , & Eloe‐Fadrosh, E. A. (2019). Spatial, Temporal, and Phylogenetic Scales of Microbial Ecology. Trends in Microbiology, 27(8), 662–669. 10.1016/j.tim.2019.03.003 31000488

[mec15538-bib-0028] Leibold, M. A. , Holyoak, M. , Mouquet, N. , Amarasekare, P. , Chase, J. M. , Hoopes, M. F. , … Gonzalez, A. (2004). The metacommunity concept: A framework for multi‐scale community ecology. Ecology Letters, 7(7), 601–613. 10.1111/j.1461-0248.2004.00608.x

[mec15538-bib-0029] Li, J. , Zhang, J. , Liu, L. , Fan, Y. , Li, L. , Yang, Y. , … Zhang, X. (2015). Annual periodicity in planktonic bacterial and archaeal community composition of eutrophic Lake Taihu. Scientific Reports, 5, 15488 10.1038/srep15488 26503553 PMC4621408

[mec15538-bib-0030] Linz, A. M. , Crary, B. C. , Shade, A. , Owens, S. , Gilbert, J. A. , Knight, R. , & McMahon, K. D. (2017). Bacterial community composition and dynamics spanning five years in freshwater bog lakes. mSphere, 2(3), e00169–e217. 10.1128/mSphere.00169-17 28680968 PMC5489657

[mec15538-bib-0031] Liu, W. , Graham, E. B. , Zhong, L. , Zhang, J. , Li, S. , Lin, X. , & Feng, Y. (2020). Long‐term stochasticity combines with short‐term variability in assembly processes to underlie rice paddy sustainability. Frontiers in Microbiology, 11, 873 10.3389/fmicb.2020.00873 32499764 PMC7243440

[mec15538-bib-0032] Llames, M. E. , Huber, P. , Metz, S. , & Unrein, F. (2017). Interplay between stochastic and deterministic processes in the maintenance of alternative community states in Verrucomicrobia‐dominated shallow lakes. FEMS Microbiology Ecology, 93(7), fix077 10.1093/femsec/fix077 28582516

[mec15538-bib-0033] Llirós, M. , Inceoğlu, Ö. , García‐Armisen, T. , Anzil, A. , Leporcq, B. , Pigneur, L.‐M. , … Servais, P. (2014). Bacterial community composition in three freshwater reservoirs of different alkalinity and trophic status. PLoS One, 9(12), e116145 10.1371/journal.pone.0116145 25541975 PMC4277477

[mec15538-bib-0034] Logares, R. , Deutschmann, I. M. , Junger, P. C. , Giner, C. R. , Krabberød, A. K. , Schmidt, T. S. B. , … Massana, R. (2020). Disentangling the mechanisms shaping the surface ocean microbiota. Microbiome, 8, 55 10.1186/s40168-020-00827-8 32312331 PMC7171866

[mec15538-bib-0035] Logares, R. , Tesson, S. V. M. , Canbäck, B. , Pontarp, M. , Hedlund, K. , & Rengefors, K. (2018). Contrasting prevalence of selection and drift in the community structuring of bacteria and microbial eukaryotes. Environmental Microbiology, 20(6), 2231–2240. 10.1111/1462-2920.14265 29727053

[mec15538-bib-0036] Lorenzen, C. J. (1967). Determination of chlorophyll and pheo‐pigments: Spectrophotometric equations1. Limnology and Oceanography, 12(2), 343–346. 10.4319/lo.1967.12.2.0343

[mec15538-bib-0037] Love, M. I. , Huber, W. , & Anders, S. (2014). Moderated estimation of fold change and' ' dispersion for RNA‐seq data with DESeq2. Genome Biology, 15, 550 10.1186/s13059-014-0550-8 25516281 PMC4302049

[mec15538-bib-0038] McCarthy, A. , Chiang, E. , Schmidt, M. L. , & Denef, V. J. (2015). RNA preservation agents and nucleic acid extraction method bias perceived bacterial community composition. PLoS One, 10(3), e0121659 10.1371/journal.pone.0121659 25798612 PMC4370824

[mec15538-bib-0039] Newton, R. J. , Jones, S. E. , Eiler, A. , McMahon, K. D. , & Bertilsson, S. (2011). A guide to the natural history of freshwater lake bacteria. Microbiology and Molecular Biology Reviews, 75(1), 14–49. 10.1128/MMBR.00028-10 21372319 PMC3063352

[mec15538-bib-0040] Niedrist, G. H. , Psenner, R. , & Sommaruga, R. (2018). Climate warming increases vertical and seasonal water temperature differences and inter‐annual variability in a mountain lake. Climatic Change, 151, 473–490. 10.1007/s10584-018-2328-6

[mec15538-bib-0041] Obertegger, U. , Bertilsson, S. , Pindo, M. , Larger, S. , & Flaim, G. (2018). Temporal variability of bacterioplankton is habitat driven. Molecular Ecology, 27(21), 4322–4335. 10.1111/mec.14855 30176079

[mec15538-bib-0042] Oksanen, J. , Blanchet, F. , Kindt, R. , Legendre, P. , Minchin, P. , Simpson, G. , … Wagner, H. (2019). Vegan: Community Ecology Package. R Package Version 2.0‐7.

[mec15538-bib-0043] Parada, A. E. , Needham, D. M. , & Fuhrman, J. A. (2016). Every base matters: Assessing small subunit rRNA primers for marine microbiomes with mock communities, time series and global field samples. Environmental Microbiology, 18, 1403–1414. 10.1111/1462-2920.13023 26271760

[mec15538-bib-0044] Pérez, M. T. , & Sommaruga, R. (2011). Temporal changes in the dominance of major planktonic bacterial groups in an alpine lake: Discrepancy with their contribution to bacterial production. Aquatic Microbial Ecology, 63, 161–170. 10.3354/ame01505

[mec15538-bib-0045] Pernthaler, J. , Glöckner, F.‐O. , Unterholzner, S. , Alfreider, A. , Psenner, R. , & Amann, R. (1998). Seasonal community and population dynamics of pelagic bacteria and archaea in a high mountain lake. Applied and Environmental Microbiology, 64(11), 4299–4306. 10.1128/AEM.64.11.4299-4306.1998 9797280 PMC106642

[mec15538-bib-0046] R Core Team . (2018). R: A language and environment for statistical' ' computing. Vienna, Austria: R Foundation for Statistical Computing.

[mec15538-bib-0047] Rofner, C. , Sommaruga, R. , & Pérez, M. T. (2016). Differential utilization patterns of dissolved organic phosphorus compounds by heterotrophic bacteria in two mountain lakes. FEMS Microbiology Ecology, 92, fiw139 10.1093/femsec/fiw139 27312963 PMC4940451

[mec15538-bib-0048] Roguet, A. , Laigle, G. S. , Therial, C. , Bressy, A. , Soulignac, F. , Catherine, A. , … Lucas, F. S. (2015). Neutral community model explains the bacterial community assembly in freshwater lakes. FEMS Microbiology Ecology, 91, fiv125 10.1093/femsec/fiv125 26472576

[mec15538-bib-0049] Ruiz‐González, C. , Garcia‐Chaves, M. C. , Ferrera, I. , Niño‐García, J. P. , & Del Giorgio, P. A. (2020). Taxonomic differences shape the responses of freshwater aerobic anoxygenic phototrophic bacterial communities to light and predation. Molecular Ecology, 29(7), 1267–1283. 10.1111/mec.15404 32147876

[mec15538-bib-0050] Rusak, J. A. , Jones, S. E. , Kent, A. D. , Shade, A. L. , & McMahon, T. M. (2009). Spatial synchrony in microbial community dynamics: Testing among‐year and lake patterns. SIL Proceedings, 1922–2010(30), 936–940. 10.1080/03680770.2009.11902275

[mec15538-bib-0051] Salcher, M. M. , Pernthaler, J. , & Posch, T. (2010). Spatiotemporal distribution and activity patterns of bacteria from three phylogenetic groups in an oligomesotrophic lake. Limnology and Oceanography, 55(2), 846–856. 10.4319/lo.2010.55.2.0846

[mec15538-bib-0052] Salka, I. , Cuperová, Z. , Mašín, M. , Koblížek, M. , & Grossart, H.‐P. (2011). Rhodoferax‐related pufM gene cluster dominates the aerobic anoxygenic phototrophic communities in German freshwater lakes. Environmental Microbiology, 13(11), 2865–2875. 10.1111/j.1462-2920.2011.02562.x 21895915

[mec15538-bib-0053] Salmaso, N. , Albanese, D. , Capelli, C. , Boscaini, A. , Pindo, M. , & Donati, C. (2018). Diversity and cyclical seasonal transitions in the bacterial community in a large and deep perialpine lake. Microbial Ecology, 76(1), 125–143. 10.1007/s00248-017-1120-x 29192335

[mec15538-bib-0054] Schauer, M. , & Hahn, M. W. (2005). Diversity and phylogenetic affiliations of morphologically conspicuous large filamentous bacteria occurring in the pelagic zones of a broad spectrum of freshwater habitats. Applied and Environmental Microbiology, 71(4), 1931–1940. 10.1128/AEM.71.4.1931-1940.2005 15812022 PMC1082555

[mec15538-bib-0055] Schütte, U. M. E. , Cadieux, S. B. , Hemmerich, C. , Pratt, L. M. , & White, J. R. (2016). Unanticipated Geochemical and Microbial Community Structure under Seasonal Ice Cover in a Dilute, Dimictic Arctic Lake. Frontiers in Microbiology, 7, 1035 10.3389/fmicb.2016.01035. eCollection 2016 27458438 PMC4932660

[mec15538-bib-0056] Segata, N. , Izard, J. , Waldron, L. , Gevers, D. , Miropolsky, L. , Garrett, W. S. , & Huttenhower, C. (2011). Metagenomic biomarker discovery and explanation. Genome Biology, 12(6), R60 10.1186/gb-2011-12-6-r60 21702898 PMC3218848

[mec15538-bib-0057] Shade, A. , Caporaso, J. G. , Handelsman, J. , Knight, R. , & Fierer, N. (2013). A meta‐analysis of changes in bacterial and archaeal communities with time. The ISME Journal, 7(8), 1493–1506. 10.1038/ismej.2013.54 23575374 PMC3721121

[mec15538-bib-0058] Shade, A. , Jones, S. E. , & McMahon, K. D. (2008). The influence of habitat heterogeneity on freshwater bacterial community composition and dynamics. Environmental Microbiology, 10(4), 1057–1067. 10.1111/j.1462-2920.2007.01527.x 18218031

[mec15538-bib-0059] Shade, A. , Kent, A. D. , Jones, S. E. , Newton, R. J. , Triplett, E. W. , & McMahon, K. D. (2007). Interannual dynamics and phenology of bacterial communities in a eutrophic lake. Limnology and Oceanography, 52(2), 487–494. 10.4319/lo.2007.52.2.0487

[mec15538-bib-0060] Sommaruga, R. , & Psenner, R. (1995). Permanent presence of grazing‐resistant bacteria in a hypertrophic lake. Applied and Environmental Microbiology, 61(9), 3457–3459. 10.1128/AEM.61.9.3457-3459.1995 16535131 PMC1388585

[mec15538-bib-0061] Stegen, J. C. , Lin, X. , Fredrickson, J. K. , Chen, X. , Kennedy, D. W. , Murray, C. J. , … Konopka, A. (2013). Quantifying community assembly processes and identifying features that impose them. The ISME Journal, 7(11), 2069–2079. 10.1038/ismej.2013.93 23739053 PMC3806266

[mec15538-bib-0062] Stegen, J. C. , Lin, X. , Fredrickson, J. K. , & Konopka, A. E. (2015). Estimating and mapping ecological processes influencing microbial community assembly. Frontiers in Microbiology, 6, 370 10.3389/fmicb.2015.00370 25983725 PMC4416444

[mec15538-bib-0063] Stegen, J. C. , Lin, X. , Konopka, A. E. , & Fredrickson, J. K. (2012). Stochastic and deterministic assembly processes in subsurface microbial communities. The ISME Journal, 6(9), 1653–1664. 10.1038/ismej.2012.22 22456445 PMC3498916

[mec15538-bib-0064] Tammert, H. , Tšertova, N. , Kiprovskaja, J. , Baty, F. , Nõges, T. , & Kisand, V. (2015). Contrasting seasonal and interannual environmental drivers in bacterial communities within a large shallow lake: Evidence from a seven year survey. Aquatic Microbial Ecology, 75, 43–54. 10.3354/ame01744

[mec15538-bib-0065] Tartarotti, B. , & Sommaruga, R. (2006). Seasonal and ontogenetic changes of mycosporine‐like amino acids in planktonic organisms from an alpine lake. Limnology and Oceanography, 51(3), 1530–1541. 10.4319/lo.2006.51.3.1530 21258624 PMC3024532

[mec15538-bib-0067] Tran, P. , Ramachandran, A. , Khawasik, O. et al (2018). Microbial life under ice: Metagenome diversity and in situ activity of Verrucomicrobia in seasonally ice‐covered Lakes. Environmental Microbiology, 20(7), 2568–2584. 10.1111/1462-2920.14283 29921005

[mec15538-bib-0068] Van der Gucht, K. , Sabbe, K. , De Meester, L. , Vloemans, N. , Zwart, G. , Gillis, M. , & Vyverman, W. (2001). Contrasting bacterioplankton community composition and seasonal dynamics in two neighbouring hypertrophic freshwater lakes. Environmental Microbiology, 3, 680–690. 10.1046/j.1462-2920.2001.00242.x 11846758

[mec15538-bib-0069] Vanwonterghem, I. , Jensen, P. D. , Dennis, P. G. , Hugenholtz, P. , Rabaey, K. , & Tyson, G. W. (2014). Deterministic processes guide long‐term synchronised population dynamics in replicate anaerobic digesters. The ISME Journal, 8, 2015–2028. 10.1038/ismej.2014.50 24739627 PMC4184015

[mec15538-bib-0070] Vass, M. , Székely, A. J. , Lindström, E. S. , & Langenheder, S. (2020). Using null models to compare bacterial and microeukaryotic metacommunity assembly under shifting environmental conditions. Scientific Reports, 10, 2455 10.1038/s41598-020-59182-1 32051469 PMC7016149

[mec15538-bib-0071] Vellend, M. (2010). Conceptual synthesis in community ecology. The Quarterly Review of Biology, 85(2), 183–206. 10.1086/652373 20565040

[mec15538-bib-0079] Vogler, P. (1966). Zur Analytik der Phosphorverbindungen in Gewässer. Limnologica, 4, 437–444.

[mec15538-bib-0082] Wagner, R. (1969). Neue Aspekte zur Stickstoffanalytik in der Wasserchemie. Vom Wasser, 36, 263–318.

[mec15538-bib-0072] Wang, J. , Shen, J. I. , Wu, Y. , Tu, C. , Soininen, J. , Stegen, J. C. , … Zhang, E. (2013). Phylogenetic beta diversity in bacterial assemblages across ecosystems: Deterministic versus stochastic processes. The ISME Journal, 7(7), 1310–1321. 10.1038/ismej.2013.30 23446837 PMC3695296

[mec15538-bib-0073] Wille, A. , Sonntag, B. , Sattler, B. , & Psenner, R. (1999). Abundance, biomass and size structure of the microbial assemblage in the high mountain lake Gossenköllesee (Tyrol, Austria) during the ice‐free period. Journal of Limnology, 58(2), 117 10.4081/jlimnol.1999.117

[mec15538-bib-0074] Yan, Q. , Stegen, J. C. , Yu, Y. , Deng, Y. E. , Li, X. , Wu, S. , … Zhou, J. (2017). Nearly a decade‐long repeatable seasonal diversity patterns of bacterioplankton communities in the eutrophic Lake Donghu (Wuhan, China). Molecular Ecology, 26(14), 3839–3850. 10.1111/mec.14151 28437572

[mec15538-bib-0075] Yannarell, A. C. , Kent, A. D. , Lauster, G. H. , Kratz, T. K. , & Triplett, E. W. (2003). Temporal patterns in bacterial communities in three temperate lakes of different trophic status. Microbial Ecology, 46, 391–405. 10.1007/s00248-003-1008-9 12904915

[mec15538-bib-0076] Yannarell, A. C. , & Triplett, E. W. (2005). Geographic and environmental sources of variation in lake bacterial community composition. Applied and Environmental Microbiology, 71(1), 227–239. 10.1128/AEM.71.1.227-239.2005 15640192 PMC544217

[mec15538-bib-0077] Zhao, D. , Cao, X. , Huang, R. , Zeng, J. , Shen, F. , Xu, H. , … Yu, Z. (2017). The heterogeneity of composition and assembly processes of the microbial community between different nutrient loading lake zones in Taihu Lake. Applied Microbiology and Biotechnology, 101(14), 5913–5923. 10.1007/s00253-017-8327-0 28523397

[mec15538-bib-0078] Zhou, J. , & Ning, D. (2017). Stochastic community assembly: Does it matter in microbial ecology? Microbiology and Molecular Biology Reviews, 81, e00002 ‐17 10.1128/MMBR.00002-17 29021219 PMC5706748

