# Supplementary material for: The balance between deterministic and stochastic processes in structuring lake bacterioplankton community over time
Source: Mol Ecol. 2020 Jul 24;29(16):3117–30. doi: 10.1111/mec.15538 (PMC7540538; doi:10.1111/mec.15538)

**Supplemental Information**

**The balance between deterministic and stochastic processes in structuring lake bacterioplankton community over time**

***Pablo Aguilar and Ruben Sommaruga***

Lake and Glacier Ecology Research Group, Department of Ecology, University of Innsbruck, Technikerstr. 25, 6020 Innsbruck, Austria.

Correspondence: Pablo Aguilar, Department of Ecology, University of Innsbruck, Austria. Email: pabloaguilar.e@gmail.com or Ruben Sommaruga, Department of Ecology, University of Innsbruck, Innsbruck, Austria. Email: ruben.sommaruga@uibk.ac.at

**Table S1** Taxonomic features identified as statistically significantly different between Gossenköllesee (GKS) and Piburgersee (PIB).

| **feature** | **Lake** | **LDA score (log10)** | **Significance** | **Phylum** | **Class** | **Order** | **Family** | **Genus** |
| --- | --- | --- | --- | --- | --- | --- | --- | --- |
| 1 | GKS | -3.764982942 | 0.035134202 | Proteobacteria | Alphaproteobacteria | Acetobacterales | Acetobacteraceae |  |
| 2 | GKS | -3.937311119 | 0.039067845 | Proteobacteria | Alphaproteobacteria | Rhizobiales | Xanthobacteraceae |  |
| 3 | GKS | -5.164185065 | 7.77384E-13 | Proteobacteria | Gammaproteobacteria | Betaproteobacteriales | Burkholderiaceae | Polaromonas |
| 4 | GKS | -4.75245139 | 1.45815E-05 | Actinobacteria | Actinobacteria | Micrococcales | Microbacteriaceae |  |
| 5 | GKS | -5.093194404 | 1.12581E-10 | Proteobacteria | Gammaproteobacteria | Betaproteobacteriales | Burkholderiaceae |  |
| 6 | GKS | -4.744783903 | 0.003903904 | Proteobacteria |  |  |  |  |
| 7 | GKS | -3.692179424 | 0.049955353 | Bacteroidetes | Bacteroidia | Cytophagales | Spirosomaceae | Lacihabitans |
| 8 | GKS | -4.745741152 | 0.002410841 | Proteobacteria | Gammaproteobacteria |  |  |  |
| 9 | GKS | -4.866046847 | 2.35601E-07 | Actinobacteria |  |  |  |  |
| 10 | GKS | -3.533787662 | 0.020535715 | Firmicutes | Clostridia | Clostridiales | Clostridiaceae_1 |  |
| 11 | GKS | -3.972153335 | 0.012065755 | Actinobacteria | Actinobacteria | Corynebacteriales |  |  |
| 12 | GKS | -4.84235516 | 6.60302E-07 | Actinobacteria | Actinobacteria |  |  |  |
| 13 | GKS | -3.854478979 | 0.049955353 | Actinobacteria | Actinobacteria | Corynebacteriales | Nocardiaceae |  |
| 14 | GKS | -3.857715898 | 0.049955353 | Actinobacteria | Actinobacteria | Corynebacteriales | Nocardiaceae | Rhodococcus |
| 15 | GKS | -3.583522803 | 0.049955353 | Actinobacteria | Acidimicrobiia | Microtrichales |  |  |
| 16 | GKS | -4.727345981 | 0.004096507 | Proteobacteria | Gammaproteobacteria | Betaproteobacteriales |  |  |
| 17 | GKS | -3.676494928 | 0.024672403 | Actinobacteria | Acidimicrobiia |  |  |  |
| 18 | GKS | -4.763957134 | 1.26547E-05 | Actinobacteria | Actinobacteria | Micrococcales |  |  |
| 19 | GKS | -4.845811013 | 2.87172E-09 | Actinobacteria | Actinobacteria | Micrococcales | Microbacteriaceae | Frondihabitans |
| 20 | GKS | -3.776592845 | 0.024672403 | Proteobacteria | Alphaproteobacteria | Acetobacterales |  |  |
| 21 | PIB | 3.113680204 | 0.047737821 | Proteobacteria | Deltaproteobacteria | Myxococcales | Polyangiaceae | NA |
| 22 | PIB | 3.09947209 | 0.047737821 | Firmicutes | Clostridia | NA | NA |  |
| 23 | PIB | 3.709284993 | 0.004290489 | Proteobacteria | Deltaproteobacteria | Bdellovibrionales |  |  |
| 24 | PIB | 3.081051086 | 0.047737821 | Tenericutes | Mollicutes | Mollicutes_RF39 | NA |  |
| 25 | PIB | 3.426633304 | 0.01479904 | Firmicutes | Bacilli | Bacillales | Paenibacillaceae |  |
| 26 | PIB | 3.859097857 | 1.12564E-06 | Caldiserica | Caldisericia | Caldisericales | TTA_B15 |  |
| 27 | PIB | 3.083424571 | 0.047737821 | Cyanobacteria | Oxyphotobacteria | Nostocales | Phormidiaceae | Tychonema_CCAP_1459_11B |
| 28 | PIB | 3.793816499 | 1.22808E-05 | Proteobacteria | Deltaproteobacteria | Syntrophobacterales |  |  |
| 29 | PIB | 4.155574977 | 1.62107E-09 | Proteobacteria | Alphaproteobacteria | Rhizobiales | Rhizobiales_Incertae_Sedis | Alsobacter |
| 30 | PIB | 3.687436413 | 0.00046289 | Proteobacteria | Gammaproteobacteria | Betaproteobacteriales | Rhodocyclaceae | Sterolibacterium |
| 31 | PIB | 3.733355545 | 0.000672613 | Proteobacteria | Gammaproteobacteria | Betaproteobacteriales | Burkholderiaceae | Paucibacter |
| 32 | PIB | 3.285947741 | 0.047737821 | Chloroflexi | Chloroflexia | Chloroflexales | Chloroflexaceae |  |
| 33 | PIB | 3.401037944 | 0.047737821 | Bacteroidetes | Chlorobia | Chlorobiales | Chlorobiaceae | Chlorobium |
| 34 | PIB | 3.798772564 | 0.00046289 | Firmicutes | Clostridia | Clostridiales | Lachnospiraceae |  |
| 35 | PIB | 2.984571944 | 0.01479904 | Firmicutes | Erysipelotrichia | Erysipelotrichales |  |  |
| 36 | PIB | 2.976269686 | 0.047737821 | Planctomycetes | Phycisphaerae | Phycisphaerales | AKAU3564_sediment_group |  |
| 37 | PIB | 4.391909726 | 5.67034E-14 | Chloroflexi | SL56_marine_group |  |  |  |
| 38 | PIB | 4.229816436 | 1.18513E-08 | Planctomycetes | Planctomycetacia | Pirellulales |  |  |
| 39 | PIB | 3.709799813 | 0.001475058 | Proteobacteria | Gammaproteobacteria | Betaproteobacteriales | Hydrogenophilaceae |  |
| 40 | PIB | 3.142909986 | 0.047737821 | Epsilonbacteraeota | Campylobacteria | Campylobacterales | Thiovulaceae | Sulfurimonas |
| 41 | PIB | 3.22720807 | 0.047737821 | Proteobacteria | Gammaproteobacteria | Betaproteobacteriales | Burkholderiaceae | Polynucleobacter |
| 42 | PIB | 3.836081839 | 0.00046289 | Firmicutes | Clostridia | Clostridiales | Ruminococcaceae | Candidatus_Soleaferrea |
| 43 | PIB | 3.703779669 | 4.39004E-05 | Proteobacteria | Gammaproteobacteria | Betaproteobacteriales | Burkholderiaceae | GKS98_freshwater_group |
| 44 | PIB | 3.322774353 | 0.01479904 | Bacteroidetes | Bacteroidia | Sphingobacteriales | ST_12K33 | NA |
| 45 | PIB | 4.01662461 | 6.29625E-09 | Epsilonbacteraeota |  |  |  |  |
| 46 | PIB | 4.019458418 | 1.04916E-05 | Bacteroidetes | Bacteroidia | Flavobacteriales |  |  |
| 47 | PIB | 3.247156208 | 0.047737821 | Bacteroidetes | Bacteroidia | Cytophagales | Amoebophilaceae |  |
| 48 | PIB | 2.960743827 | 0.047737821 | Proteobacteria | Deltaproteobacteria | Oligoflexales | 053A03_B_DI_P58 | NA |
| 49 | PIB | 4.331330845 | 2.32103E-10 | Bacteroidetes | Bacteroidia | Bacteroidales |  |  |
| 50 | PIB | 3.114596479 | 0.047737821 | Firmicutes | Clostridia | NA |  |  |
| 51 | PIB | 4.011370828 | 2.38514E-08 | Proteobacteria | Deltaproteobacteria | Bradymonadales | NA | NA |
| 52 | PIB | 3.009195719 | 0.047737821 | Chloroflexi | Dehalococcoidia | MSBL5 | NA |  |
| 53 | PIB | 4.272464562 | 4.06754E-10 | Lentisphaerae | Oligosphaeria | Oligosphaerales |  |  |
| 54 | PIB | 3.09494497 | 0.01479904 | Acidobacteria | Acidobacteriia | Solibacterales | Solibacteraceae__Subgroup_3_ | Candidatus_Solibacter |
| 55 | PIB | 3.308648925 | 0.01479904 | Bacteroidetes | Bacteroidia | Sphingobacteriales | ST_12K33 |  |
| 56 | PIB | 3.243889462 | 0.047737821 | Fusobacteria | Fusobacteriia | Fusobacteriales |  |  |
| 57 | PIB | 3.639646144 | 0.000466852 | Cyanobacteria | Melainabacteria |  |  |  |
| 58 | PIB | 3.299422441 | 0.047737821 | Cloacimonetes | Cloacimonadia |  |  |  |
| 59 | PIB | 3.343795968 | 0.01479904 | Bacteroidetes | Bacteroidia | Bacteroidales | FTLpost3 |  |
| 60 | PIB | 2.986845657 | 0.01479904 | Chloroflexi | Dehalococcoidia | vadinBA26 |  |  |
| 61 | PIB | 3.167803934 | 0.047737821 | Proteobacteria | Alphaproteobacteria | Rickettsiales | Midichloriaceae | MD3_55 |
| 62 | PIB | 3.203937586 | 0.047737821 | Fusobacteria |  |  |  |  |
| 63 | PIB | 3.274180808 | 0.01479904 | Lentisphaerae | Lentisphaeria | Victivallales | Victivallaceae |  |
| 64 | PIB | 4.318321917 | 4.82073E-11 | Proteobacteria | Gammaproteobacteria | Betaproteobacteriales | Nitrosomonadaceae |  |
| 65 | PIB | 3.561109743 | 0.0001436 | Patescibacteria | ABY1 | Candidatus_Uhrbacteria | NA | NA |
| 66 | PIB | 4.018168 | 6.29625E-09 | Epsilonbacteraeota | Campylobacteria | Campylobacterales |  |  |
| 67 | PIB | 4.391909814 | 5.67034E-14 | Chloroflexi | SL56_marine_group | NA | NA | NA |
| 68 | PIB | 2.910413326 | 0.047737821 | Planctomycetes | Phycisphaerae | Phycisphaerales | AKAU3564_sediment_group | NA |
| 69 | PIB | 3.099269693 | 0.047737821 | Firmicutes | Clostridia | NA | NA | NA |
| 70 | PIB | 3.878662065 | 1.12564E-06 | Caldiserica | Caldisericia | Caldisericales |  |  |
| 71 | PIB | 3.020096541 | 0.047737821 | Patescibacteria | Gracilibacteria | Candidatus_Peregrinibacteria | NA |  |
| 72 | PIB | 3.555193289 | 0.0001436 | Patescibacteria | ABY1 | Candidatus_Uhrbacteria |  |  |
| 73 | PIB | 3.245420266 | 0.038389777 | Bacteroidetes | Bacteroidia | Chitinophagales | Chitinophagaceae |  |
| 74 | PIB | 2.982041484 | 0.01479904 | Firmicutes | Erysipelotrichia | Erysipelotrichales | Erysipelotrichaceae |  |
| 75 | PIB | 3.234235173 | 0.047737821 | Latescibacteria | Latescibacteria |  |  |  |
| 76 | PIB | 2.967751719 | 0.047737821 | Proteobacteria | Deltaproteobacteria | Desulfobacterales | Desulfobulbaceae |  |
| 77 | PIB | 3.256591708 | 0.004669001 | Spirochaetes | Spirochaetia | Spirochaetales | Spirochaetaceae |  |
| 78 | PIB | 3.603485733 | 4.39004E-05 | Chloroflexi | Anaerolineae | Anaerolineales |  |  |
| 79 | PIB | 3.063240944 | 0.047737821 | Bacteroidetes | Bacteroidia | Bacteroidales | Paludibacteraceae |  |
| 80 | PIB | 2.948774865 | 0.047737821 | Proteobacteria | Alphaproteobacteria | Rhizobiales | NA |  |
| 81 | PIB | 2.963495243 | 0.047737821 | Bacteroidetes | Ignavibacteria | Kryptoniales |  |  |
| 82 | PIB | 4.391909602 | 5.67034E-14 | Chloroflexi | SL56_marine_group | NA | NA |  |
| 83 | PIB | 3.394605878 | 0.004669001 | Firmicutes | Clostridia | Clostridiales | Clostridiales_vadinBB60_group | NA |
| 84 | PIB | 3.504853422 | 0.047737821 | Firmicutes | Clostridia | Clostridiales | Lachnospiraceae | Natranaerovirga |
| 85 | PIB | 3.55242207 | 0.0001436 | Patescibacteria | ABY1 | Candidatus_Uhrbacteria | NA |  |
| 86 | PIB | 3.232272435 | 0.047737821 | Latescibacteria | Latescibacteria | Latescibacterales | Latescibacteraceae | Candidatus_Latescibacter |
| 87 | PIB | 3.03405905 | 0.01479904 | Proteobacteria | Alphaproteobacteria | Azospirillales | Azospirillaceae | NA |
| 88 | PIB | 3.087451418 | 0.047737821 | Actinobacteria | Actinobacteria | Micrococcales | Microbacteriaceae | NA |
| 89 | PIB | 4.055097387 | 9.16862E-07 | Bacteroidetes | Bacteroidia | Flavobacteriales | Cryomorphaceae |  |
| 90 | PIB | 3.750808977 | 4.39004E-05 | Proteobacteria | Deltaproteobacteria | Bdellovibrionales | Bacteriovoracaceae |  |
| 91 | PIB | 3.067985464 | 0.047737821 | Actinobacteria | Actinobacteria | PeM15 | NA | NA |
| 92 | PIB | 3.022372202 | 0.047737821 | Cyanobacteria | Oxyphotobacteria | Nostocales | Phormidiaceae | NA |
| 93 | PIB | 4.225509908 | 4.77707E-05 | Firmicutes |  |  |  |  |
| 94 | PIB | 3.815025656 | 1.12564E-06 | Epsilonbacteraeota | Campylobacteria | Campylobacterales | Arcobacteraceae |  |
| 95 | PIB | 4.285128853 | 4.06754E-10 | Lentisphaerae | Oligosphaeria |  |  |  |
| 96 | PIB | 3.270523574 | 0.010131426 | Lentisphaerae | Lentisphaeria | Victivallales |  |  |
| 97 | PIB | 3.285051827 | 0.047737821 | Cloacimonetes |  |  |  |  |
| 98 | PIB | 2.964215178 | 0.047737821 | Proteobacteria | Deltaproteobacteria | Oligoflexales | 053A03_B_DI_P58 |  |
| 99 | PIB | 3.406028382 | 0.047737821 | Bacteroidetes | Bacteroidia | Bacteroidales | SB_5 | NA |
| 100 | PIB | 3.007699696 | 0.047737821 | Proteobacteria | Alphaproteobacteria | Caedibacterales | Caedibacteraceae | Caedibacter |
| 101 | PIB | 3.331638866 | 0.00046289 | Epsilonbacteraeota | Campylobacteria | Campylobacterales | NA | NA |
| 102 | PIB | 3.206342038 | 0.047737821 | Latescibacteria | Latescibacteria | Latescibacterales |  |  |
| 103 | PIB | 3.090518524 | 0.047737821 | Tenericutes | Mollicutes |  |  |  |
| 104 | PIB | 2.982655353 | 0.047737821 | Chloroflexi | Anaerolineae | NA |  |  |
| 105 | PIB | 4.217955073 | 4.06754E-10 | Planctomycetes | Planctomycetacia | Pirellulales | Pirellulaceae | Pir3_lineage |
| 106 | PIB | 3.435101208 | 0.047737821 | Bacteroidetes | Bacteroidia | Bacteroidales | SB_5 |  |
| 107 | PIB | 3.400476192 | 0.01479904 | Proteobacteria | Gammaproteobacteria | Betaproteobacteriales | Nitrosomonadaceae | GOUTA6 |
| 108 | PIB | 3.604427104 | 0.0001436 | Proteobacteria | Gammaproteobacteria | Betaproteobacteriales | Gallionellaceae | Candidatus_Nitrotoga |
| 109 | PIB | 4.074395446 | 6.29625E-09 | Actinobacteria | Actinobacteria | Micrococcales | Microbacteriaceae | Candidatus_Limnoluna |
| 110 | PIB | 3.217808315 | 0.047737821 | Latescibacteria | Latescibacteria | Latescibacterales | Latescibacteraceae |  |
| 111 | PIB | 3.071184088 | 0.047737821 | Chloroflexi | Dehalococcoidia | MSBL5 | NA | NA |
| 112 | PIB | 2.966717227 | 0.01479904 | Firmicutes | Erysipelotrichia |  |  |  |
| 113 | PIB | 3.849969808 | 0.001475058 | Proteobacteria | Alphaproteobacteria | Rickettsiales | Midichloriaceae |  |
| 114 | PIB | 3.348289606 | 0.047737821 | Bacteroidetes | Chlorobia |  |  |  |
| 115 | PIB | 3.018548351 | 0.047737821 | Bacteroidetes | Bacteroidia | Chitinophagales | Saprospiraceae | Haliscomenobacter |
| 116 | PIB | 3.480952124 | 0.009610219 | Cyanobacteria | Oxyphotobacteria | Nostocales | Microcystaceae |  |
| 117 | PIB | 3.053973463 | 0.047737821 | Firmicutes | Clostridia | Clostridiales | NA |  |
| 118 | PIB | 3.499403939 | 0.001475058 | Firmicutes | Clostridia | Clostridiales | Clostridiaceae_1 | Clostridium_sensu_stricto_9 |
| 119 | PIB | 4.479470031 | 4.12885E-12 | Chloroflexi |  |  |  |  |
| 120 | PIB | 4.016564043 | 6.29625E-09 | Epsilonbacteraeota | Campylobacteria |  |  |  |
| 121 | PIB | 4.222623689 | 1.62107E-09 | Bacteroidetes | Bacteroidia | Bacteroidales | Dysgonomonadaceae |  |
| 122 | PIB | 4.243869314 | 1.04453E-06 | Firmicutes | Clostridia |  |  |  |
| 123 | PIB | 3.246686327 | 0.004669001 | Spirochaetes | Spirochaetia | Spirochaetales |  |  |
| 124 | PIB | 3.695647096 | 1.12564E-06 | Proteobacteria | Deltaproteobacteria | Syntrophobacterales | Syntrophaceae | Desulfomonile |
| 125 | PIB | 4.082010121 | 8.81966E-08 | Bacteroidetes | Bacteroidia | Flavobacteriales | Cryomorphaceae | NA |
| 126 | PIB | 3.018191935 | 0.047737821 | Proteobacteria | Alphaproteobacteria | Rickettsiales | Rickettsiales_Incertae_Sedis |  |
| 127 | PIB | 4.564775602 | 4.05288E-05 | Bacteroidetes |  |  |  |  |
| 128 | PIB | 3.931379997 | 0.000327047 | Firmicutes | Clostridia | Clostridiales | Ruminococcaceae |  |
| 129 | PIB | 3.357069748 | 0.01479904 | Proteobacteria | Gammaproteobacteria | Betaproteobacteriales | Burkholderiaceae | Herbaspirillum |
| 130 | PIB | 3.282263954 | 0.010131426 | Lentisphaerae | Lentisphaeria |  |  |  |
| 131 | PIB | 2.986475354 | 0.01479904 | Chloroflexi | Dehalococcoidia | vadinBA26 | NA | NA |
| 132 | PIB | 3.654695359 | 1.31956E-05 | Cyanobacteria | Melainabacteria | Gastranaerophilales | NA |  |
| 133 | PIB | 4.023469227 | 0.000588608 | Cyanobacteria | Oxyphotobacteria | Nostocales |  |  |
| 134 | PIB | 3.020082981 | 0.047737821 | Cyanobacteria | Oxyphotobacteria | Nostocales | Gloeocapsaceae | Gleocapsa |
| 135 | PIB | 4.222623537 | 1.62107E-09 | Bacteroidetes | Bacteroidia | Bacteroidales | Dysgonomonadaceae | NA |
| 136 | PIB | 3.71635882 | 0.000573671 | Planctomycetes | Phycisphaerae | Tepidisphaerales |  |  |
| 137 | PIB | 4.089181644 | 8.73016E-06 | Proteobacteria | Gammaproteobacteria | Methylococcales |  |  |
| 138 | PIB | 2.970967527 | 0.047737821 | Chloroflexi | Anaerolineae | NA | NA | NA |
| 139 | PIB | 3.075569228 | 0.047737821 | Tenericutes | Mollicutes | Mollicutes_RF39 |  |  |
| 140 | PIB | 3.188580998 | 0.047737821 | Firmicutes | Clostridia | Clostridiales | Lachnospiraceae | Cellulosilyticum |
| 141 | PIB | 3.202388849 | 0.010131426 | Spirochaetes |  |  |  |  |
| 142 | PIB | 3.813968748 | 1.12564E-06 | Epsilonbacteraeota | Campylobacteria | Campylobacterales | Arcobacteraceae | Arcobacter |
| 143 | PIB | 3.055431033 | 0.047737821 | Bacteroidetes | Bacteroidia | Chitinophagales | Chitinophagaceae | Dinghuibacter |
| 144 | PIB | 3.287045114 | 0.004669001 | Bacteroidetes | Bacteroidia | Chitinophagales | Chitinophagaceae | Parasediminibacterium |
| 145 | PIB | 3.309126075 | 0.00046289 | Chloroflexi | Dehalococcoidia |  |  |  |
| 146 | PIB | 3.144134131 | 0.047737821 | Firmicutes | Clostridia | Clostridiales | Lachnospiraceae | NA |
| 147 | PIB | 3.659989967 | 1.31956E-05 | Cyanobacteria | Melainabacteria | Gastranaerophilales |  |  |
| 148 | PIB | 3.008687974 | 0.047737821 | Proteobacteria | Alphaproteobacteria | Rickettsiales | Rickettsiales_Incertae_Sedis | Candidatus_Hepatincola |
| 149 | PIB | 3.298365589 | 0.001475058 | Spirochaetes | Spirochaetia |  |  |  |
| 150 | PIB | 3.341157976 | 0.00046289 | Epsilonbacteraeota | Campylobacteria | Campylobacterales | NA |  |
| 151 | PIB | 3.057971831 | 0.047737821 | Cyanobacteria | Oxyphotobacteria | Nostocales | Gloeocapsaceae |  |
| 152 | PIB | 3.795213814 | 6.74004E-06 | Proteobacteria | Deltaproteobacteria | Syntrophobacterales | Syntrophaceae |  |
| 153 | PIB | 3.302897224 | 0.047737821 | Cloacimonetes | Cloacimonadia | Cloacimonadales |  |  |
| 154 | PIB | 3.454879794 | 0.047737821 | Kiritimatiellaeota | Kiritimatiellae |  |  |  |
| 155 | PIB | 3.075854134 | 0.004669001 | Proteobacteria | Deltaproteobacteria | Myxococcales | Polyangiaceae |  |
| 156 | PIB | 4.071776255 | 9.28724E-06 | Proteobacteria | Gammaproteobacteria | Methylococcales | Methylomonaceae |  |
| 157 | PIB | 3.493703998 | 0.047737821 | Kiritimatiellaeota |  |  |  |  |
| 158 | PIB | 3.397448687 | 0.047737821 | Bacteroidetes | Chlorobia | Chlorobiales |  |  |
| 159 | PIB | 4.341936107 | 2.96508E-10 | Lentisphaerae |  |  |  |  |
| 160 | PIB | 3.629092968 | 0.0001436 | Bacteroidetes | Bacteroidia | Cytophagales | Cyclobacteriaceae | Algoriphagus |
| 161 | PIB | 4.342672773 | 4.06754E-10 | Proteobacteria | Gammaproteobacteria | Betaproteobacteriales | Burkholderiaceae | Acidovorax |
| 162 | PIB | 3.871850303 | 1.12564E-06 | Caldiserica | Caldisericia |  |  |  |
| 163 | PIB | 4.504988261 | 8.18604E-05 | Bacteroidetes | Bacteroidia |  |  |  |
| 164 | PIB | 3.371170551 | 0.004669001 | Firmicutes | Clostridia | Clostridiales | Clostridiales_vadinBB60_group |  |
| 165 | PIB | 3.612083892 | 4.39004E-05 | Chloroflexi | Anaerolineae | Anaerolineales | Anaerolineaceae |  |
| 166 | PIB | 3.5898606 | 0.001398053 | Patescibacteria | ABY1 |  |  |  |
| 167 | PIB | 3.032160794 | 0.047737821 | Firmicutes | Clostridia | Clostridiales | Lachnospiraceae | Tyzzerella_3 |
| 168 | PIB | 3.646967357 | 4.39004E-05 | Bacteroidetes | Bacteroidia | Cytophagales | Cyclobacteriaceae |  |
| 169 | PIB | 2.952675466 | 0.047737821 | Proteobacteria | Alphaproteobacteria | Rhizobiales | NA | NA |
| 170 | PIB | 3.466678036 | 0.00046289 | Actinobacteria | Actinobacteria | Micrococcales | Microbacteriaceae | Candidatus_Aquiluna |
| 171 | PIB | 3.676792055 | 0.003025369 | Chloroflexi | Anaerolineae |  |  |  |
| 172 | PIB | 3.836418717 | 0.00046289 | Cyanobacteria | Oxyphotobacteria | Nostocales | Nostocaceae | Aphanizomenon_NIES81 |
| 173 | PIB | 3.018772811 | 0.047737821 | Patescibacteria | Gracilibacteria | Candidatus_Peregrinibacteria |  |  |
| 174 | PIB | 4.150899347 | 9.62044E-08 | Proteobacteria | Alphaproteobacteria | Rhizobiales | Rhizobiales_Incertae_Sedis |  |
| 175 | PIB | 3.878723036 | 0.012528232 | Cyanobacteria |  |  |  |  |
| 176 | PIB | 3.727487299 | 0.01479904 | Proteobacteria | Alphaproteobacteria | Rickettsiales | Midichloriaceae | Candidatus_Midichloria |
| 177 | PIB | 3.696179254 | 1.31956E-05 | Planctomycetes | Phycisphaerae | Tepidisphaerales | CPla_3_termite_group |  |
| 178 | PIB | 4.391909324 | 5.67034E-14 | Chloroflexi | SL56_marine_group | NA |  |  |
| 179 | PIB | 3.204707932 | 0.029555114 | Cyanobacteria | Oxyphotobacteria | Nostocales | Phormidiaceae |  |
| 180 | PIB | 4.694351491 | 1.10973E-20 | Proteobacteria | Gammaproteobacteria | Betaproteobacteriales | Methylophilaceae | Candidatus_Methylopumilus |
| 181 | PIB | 3.124579141 | 0.047737821 | Proteobacteria | Deltaproteobacteria | Desulfobacterales | Desulfobacteraceae | Candidatus_Desulfamplus |
| 182 | PIB | 3.268893699 | 0.047737821 | Chloroflexi | Anaerolineae | Anaerolineales | Anaerolineaceae | Anaerolinea |
| 183 | PIB | 3.277486607 | 0.047737821 | Chloroflexi | Chloroflexia | Chloroflexales | Chloroflexaceae | Candidatus_Chlorothrix |
| 184 | PIB | 2.998850622 | 0.01479904 | Chloroflexi | Dehalococcoidia | vadinBA26 | NA |  |
| 185 | PIB | 3.012201354 | 0.047737821 | Patescibacteria | Gracilibacteria | Candidatus_Peregrinibacteria | NA | NA |
| 186 | PIB | 3.055210577 | 0.01479904 | Proteobacteria | Alphaproteobacteria | Azospirillales | Azospirillaceae |  |
| 187 | PIB | 3.600116672 | 0.0001436 | Proteobacteria | Gammaproteobacteria | Betaproteobacteriales | Gallionellaceae |  |
| 188 | PIB | 4.658912088 | 3.53626E-16 | Proteobacteria | Gammaproteobacteria | Betaproteobacteriales | Methylophilaceae |  |
| 189 | PIB | 4.001764542 | 2.38514E-08 | Proteobacteria | Deltaproteobacteria | Bradymonadales |  |  |
| 190 | PIB | 3.436372225 | 0.03932902 | Bacteroidetes | Bacteroidia | Sphingobacteriales |  |  |
| 191 | PIB | 4.266264937 | 1.62107E-09 | Lentisphaerae | Oligosphaeria | Oligosphaerales | Oligosphaeraceae |  |
| 192 | PIB | 3.29990141 | 0.01479904 | Epsilonbacteraeota | Campylobacteria | Campylobacterales | Thiovulaceae |  |
| 193 | PIB | 4.326084808 | 1.22774E-12 | Proteobacteria | Gammaproteobacteria | Betaproteobacteriales | Nitrosomonadaceae | Nitrosospira |
| 194 | PIB | 3.491975192 | 0.034342966 | Patescibacteria |  |  |  |  |
| 195 | PIB | 3.833683391 | 0.00046289 | Cyanobacteria | Oxyphotobacteria | Nostocales | Nostocaceae |  |
| 196 | PIB | 3.98807936 | 3.89294E-06 | Proteobacteria | Gammaproteobacteria | Methylococcales | Methylomonaceae | Methyloglobulus |
| 197 | PIB | 3.069092753 | 0.047737821 | Firmicutes | Clostridia | Clostridiales | NA | NA |
| 198 | PIB | 3.074941383 | 0.047737821 | Actinobacteria | Actinobacteria | PeM15 | NA |  |
| 199 | PIB | 3.35927207 | 0.01479904 | Bacteroidetes | Bacteroidia | Bacteroidales | FTLpost3 | NA |
| 200 | PIB | 2.97926698 | 0.047737821 | Chloroflexi | Anaerolineae | NA | NA |  |
| 201 | PIB | 4.275927652 | 1.63944E-06 | Proteobacteria | Deltaproteobacteria |  |  |  |
| 202 | PIB | 3.882325668 | 1.12564E-06 | Caldiserica |  |  |  |  |
| 203 | PIB | 3.572724133 | 0.004669001 | Proteobacteria | Alphaproteobacteria | Rhizobiales | Beijerinckiaceae | Methylorosula |
| 204 | PIB | 3.112089386 | 0.047737821 | Bacteroidetes | Ignavibacteria | Ignavibacteriales |  |  |
| 205 | PIB | 3.267066943 | 0.01479904 | Lentisphaerae | Lentisphaeria | Victivallales | Victivallaceae | NA |
| 206 | PIB | 3.139894395 | 0.047737821 | Fusobacteria | Fusobacteriia |  |  |  |
| 207 | PIB | 3.446036003 | 0.001475058 | Cyanobacteria | Oxyphotobacteria | Nostocales | Microcystaceae | Microcystis_PCC_7914 |
| 208 | PIB | 3.008866265 | 0.047737821 | Firmicutes | Clostridia | Clostridiales | Family_XIII |  |
| 209 | PIB | 3.685879056 | 0.001475058 | Proteobacteria | Gammaproteobacteria | Betaproteobacteriales | Hydrogenophilaceae | NA |
| 210 | PIB | 4.156996589 | 0.001336208 | Planctomycetes |  |  |  |  |
| 211 | PIB | 3.701553809 | 1.31956E-05 | Planctomycetes | Phycisphaerae | Tepidisphaerales | CPla_3_termite_group | NA |
| 212 | PIB | 3.742786839 | 4.39004E-05 | Proteobacteria | Deltaproteobacteria | Bdellovibrionales | Bacteriovoracaceae | NA |
| 213 | PIB | 3.040622254 | 0.01479904 | Proteobacteria | Alphaproteobacteria | Azospirillales |  |  |
| 214 | PIB | 3.087157266 | 0.047737821 | Tenericutes |  |  |  |  |
| 215 | PIB | 3.352717809 | 0.001475058 | Bacteroidetes | Ignavibacteria |  |  |  |
| 216 | PIB | 4.199989317 | 3.40133E-06 | Firmicutes | Clostridia | Clostridiales |  |  |
| 217 | PIB | 3.854417422 | 1.12564E-06 | Caldiserica | Caldisericia | Caldisericales | TTA_B15 | NA |
| 218 | PIB | 3.781437346 | 0.000308561 | Proteobacteria | Gammaproteobacteria | Betaproteobacteriales | Rhodocyclaceae |  |
| 219 | PIB | 3.002109032 | 0.047737821 | Chloroflexi | Dehalococcoidia | MSBL5 |  |  |
| 220 | PIB | 2.955198488 | 0.047737821 | Firmicutes | Erysipelotrichia | Erysipelotrichales | Erysipelotrichaceae | Erysipelothrix |
| 221 | PIB | 4.165118213 | 1.49812E-05 | Planctomycetes | Planctomycetacia |  |  |  |
| 222 | PIB | 3.67239759 | 1.31956E-05 | Cyanobacteria | Melainabacteria | Gastranaerophilales | NA | NA |
| 223 | PIB | 4.006827909 | 2.38514E-08 | Proteobacteria | Deltaproteobacteria | Bradymonadales | NA |  |
| 224 | PIB | 3.078071377 | 0.047737821 | Tenericutes | Mollicutes | Mollicutes_RF39 | NA | NA |
| 225 | PIB | 4.266264941 | 1.62107E-09 | Lentisphaerae | Oligosphaeria | Oligosphaerales | Oligosphaeraceae | NA |
| 226 | PIB | 4.231825133 | 1.18513E-08 | Planctomycetes | Planctomycetacia | Pirellulales | Pirellulaceae |  |
| 227 | PIB | 3.080471436 | 0.047737821 | Actinobacteria | Actinobacteria | PeM15 |  |  |
| 228 | PIB | 3.364289992 | 0.047737821 | Bacteroidetes | Chlorobia | Chlorobiales | Chlorobiaceae |  |
| 229 | PIB | 3.358212595 | 0.007144979 | Bacteroidetes | Bacteroidia | Chitinophagales |  |  |
| 230 | PIB | 3.35693673 | 0.047737821 | Firmicutes | Bacilli | Bacillales | Paenibacillaceae | NA |

**Table S2** Loadings of measured environmental variables and MEM eigenvectors (Moran’s eigenvector maps) on PCA axes corresponding to the analysis for Gossenköllesee. Any loadings weaker than 0.001 are listed as null. Significant PCA axes for βNTI are shown in bold. T: water temperature. Cond: electrical conductivity. DOC: dissolved organic carbon. DN: dissolved nitrogen.

**Table S3** Loadings of measured environmental variables and MEM eigenvectors (Moran’s eigenvector maps) on PCA axes corresponding to the analysis for Piburgersee. Any loadings weaker than 0.001 are listed as null. Significant PCA axes for βNTI are shown in bold. T: water temperature. Cond: electrical conductivity. DOC: dissolved organic carbon. DN: dissolved nitrogen. Oxy: dissolved oxygen.

**Figure S1** Phylogenetic Moran's I correlogram showing significant phylogenetic signal (red line) across short phylogenetic distances for water temperature and pH in Gossenköllesee (GKS) and water temperature and dissolved oxygen in Piburgersee (PIB).

**
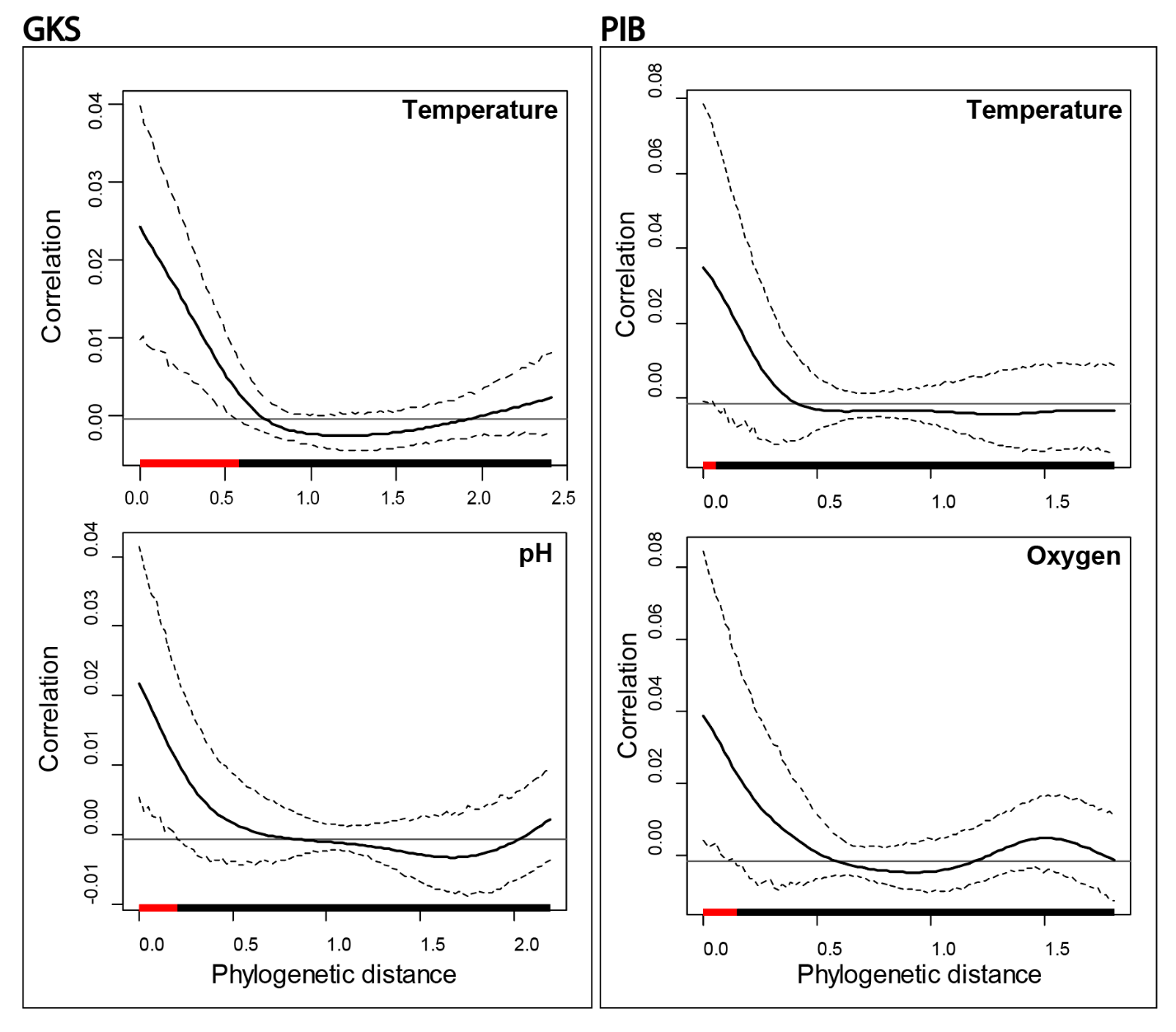
**

**Figure S2** Boxplot of environmental parameters showing significance differences between Gossenköllesee (GKS) and Piburgersee (PIB). T: water temperature. DN: dissolved nitrogen. Cond: electrical conductivity. Chl-a: chlorophyll-α. TDP: total dissolved phosphorus. DOC: dissolved organic carbon.

**
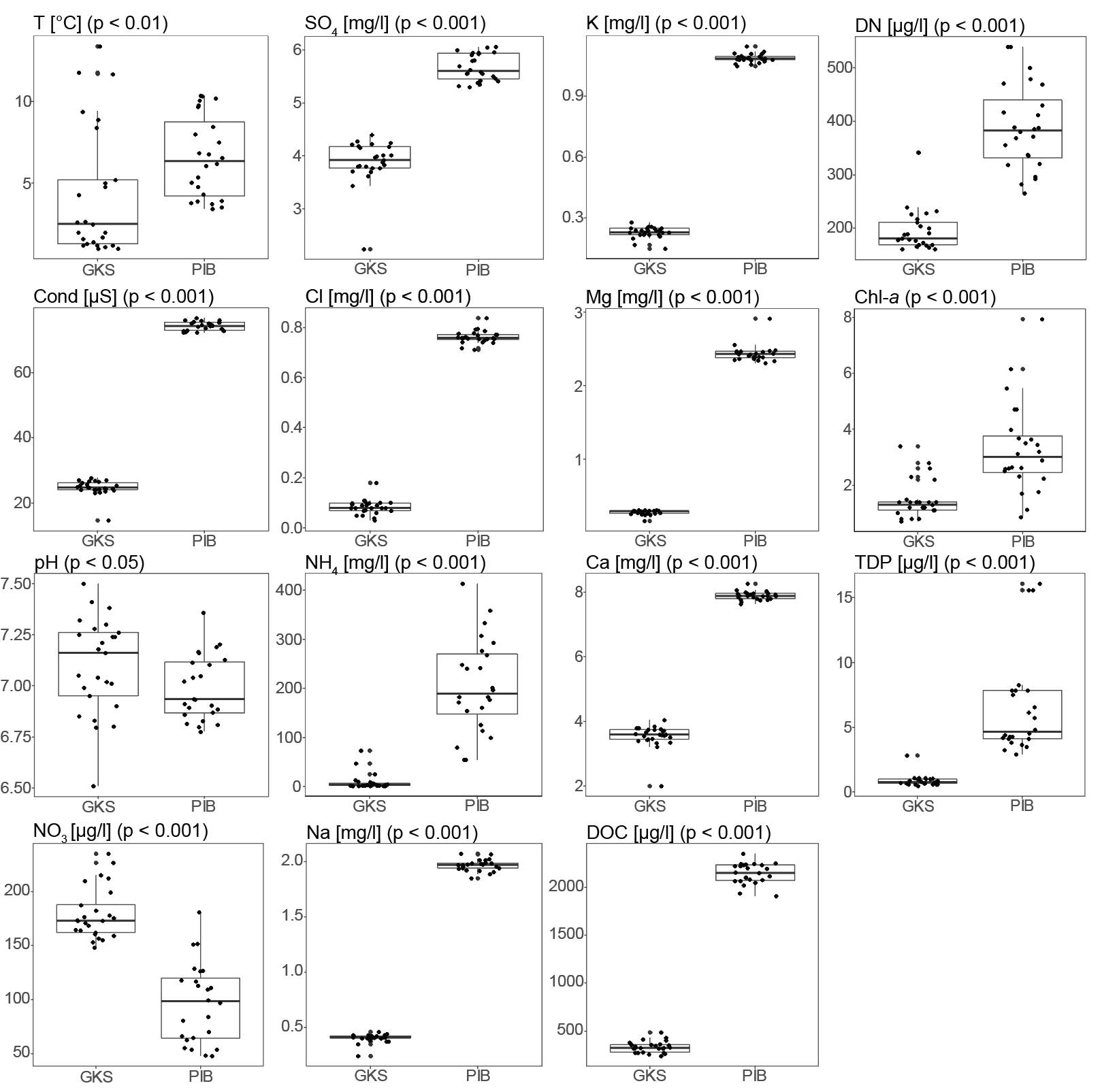
**

**Figure S3.** Temporal variability of environmental parameters in Gossenköllesee between November 2014 and December 2016 (A), in Gossenköllesee during the short-term sampling (B) and in Piburgersee between December 2014 and December 2016 (C). Values are the mean for the water column and error bars represent one standard deviation. Concentrations of DOC and DN in B were measured only once. T: water temperature. Cond: elctrical conductivity. DOC: dissolved organic carbon. DN: dissolved nitrogen. Chl-a: chlorophyll-a. TDP: total dissolved phosphorus. O: dissolved oxygen. Months in gray indicate the ice-covered period.

**
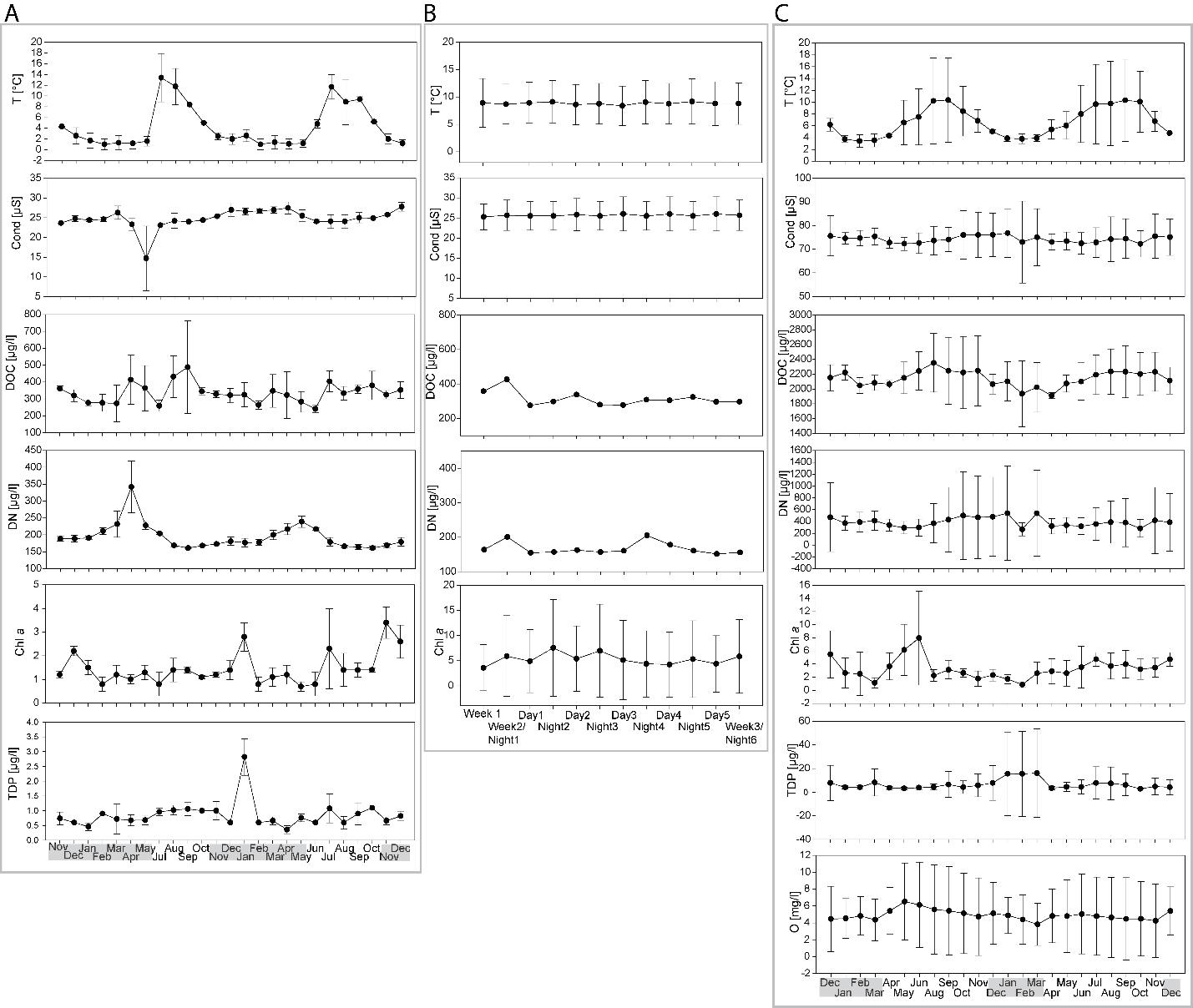
**

**Figure S4** Changes in diversity estimates during the short-term sampling in Gossenköllesee (A) and in the water column of Gossenköllesee (GKS) and Piburgersee (PIB).

**
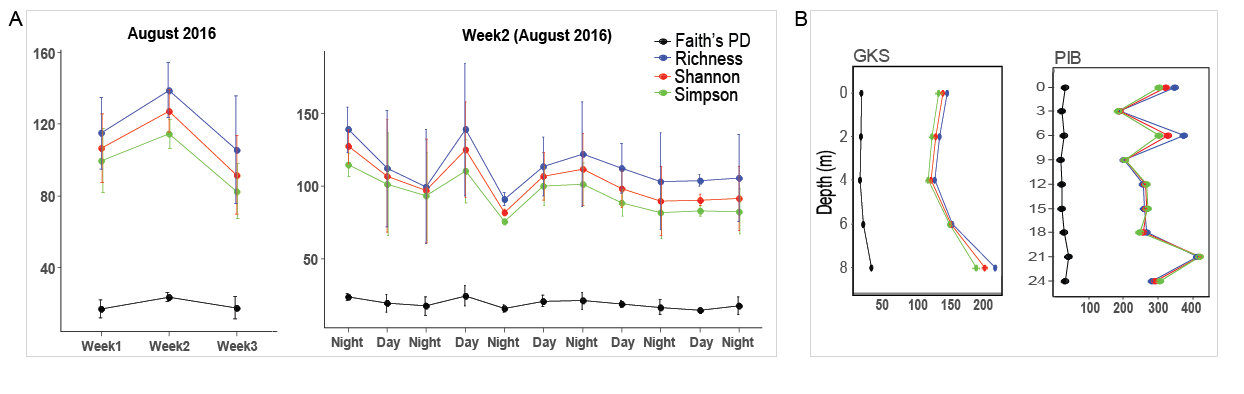
**

**Figure S5** Relative abundance (%) of bacterial phyla during the short-term sampling in Gossenköllesee (A) and in the water column of Gossenköllesee (GKS) and of Piburgersee (PIB) (B)


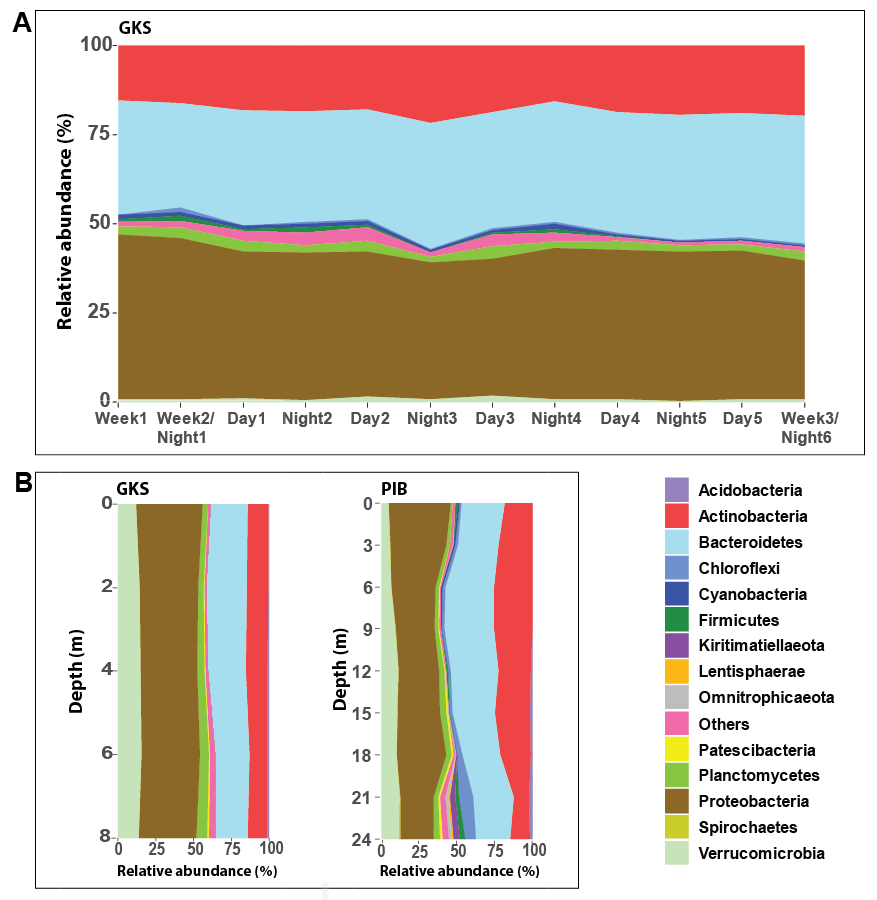


**Figure S6** Temporal changes of the most abundant genera in Gossenköllesee (GKS) and Piburgersee (PIB).


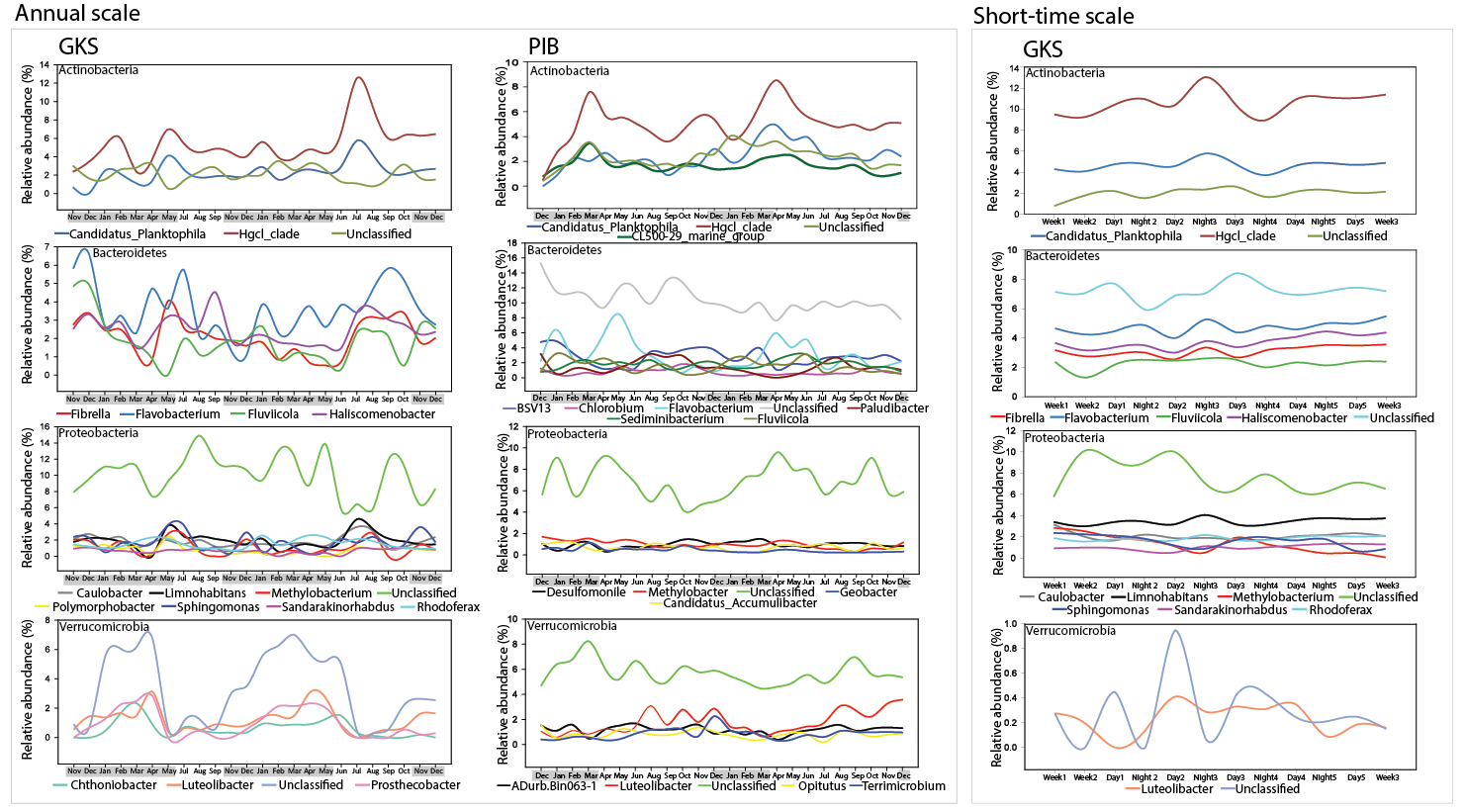

Supplement: Supplementary file 1 — Supplementary Material [file MEC-29-3117-s001.docx]
